# Biomimetic Membrane Interface Technologies for Detection and Isolation of CTCs and EVs: Advances and Opportunities in Liquid Biopsy

**DOI:** 10.1002/advs.202519127

**Published:** 2026-01-28

**Authors:** Duo Liu, Jie Yu, Jingxue Li, Yixue Chen, Mengle Peng, Yifan Xu, Yongjun Wu, Lihua Ding, Sitian He

**Affiliations:** ^1^ College of Public Health Zhengzhou University Zhengzhou China; ^2^ Puyang Center for Disease Control and Prevention Puyang China; ^3^ School of Stomatology Henan University China; ^4^ Department of Clinical Laboratory Hainan General Hospital Hainan Affiliated Hospital of Hainan Medical University Haikou China; ^5^ The Medical Laboratory Central Hainan General Hospital Hainan Affiliated Hospital of Hainan Medical University Haikou China

**Keywords:** biomimetic membrane interface, circulating tumor cells (CTCs), extracellular vesicles (EVs), liquid biopsy

## Abstract

Biomimetic membrane interface engineering constructs functionalized detection platforms by incorporating natural cell membranes, synthetic lipids, or hybrid membranes. The primary purpose of this strategy is to minimize background interference while leveraging intrinsic membrane properties for effective target interaction. Applications are diverse, ranging from the high‐purity separation of circulating tumor cells (CTCs) to the efficient isolation of extracellular vesicles (EVs), as well as the subsequent detection of EV‐derived contents (e.g., miRNA, protein, and mRNA) via membrane fusion mechanisms. Conceptually, this approach serves as a robust bridge between synthetic materials and biological systems. Its major advantages lie in the significant reduction of non‐specific binding and the unique capability to facilitate both target capture and internal cargo analysis. However, challenges such as complex preparation processes, stability issues, and the dependence on functional modifications to address significant tumor heterogeneity remain to be resolved. This review summarizes recent progress, analyzes these critical issues, and outlines future directions.

## Graphical abstract

Biomimetic membrane interface engineering constructs functionalized detection platforms by integrating natural cell membranes, synthetic lipids, or hybrid membranes. This strategy effectively reduces background interference and enables efficient target capture and analysis, showing broad applications in circulating tumor cell separation, extracellular vesicle enrichment, and cargo detection. This review summarizes recent advances, analyzes key challenges, and outlines future directions. (The illustration was generated using BioRender under a licensed subscription (Agreement number: UH295PE7TJ)).

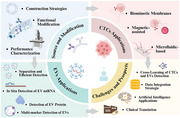

## 1 Introduction

**FIGURE 1:**
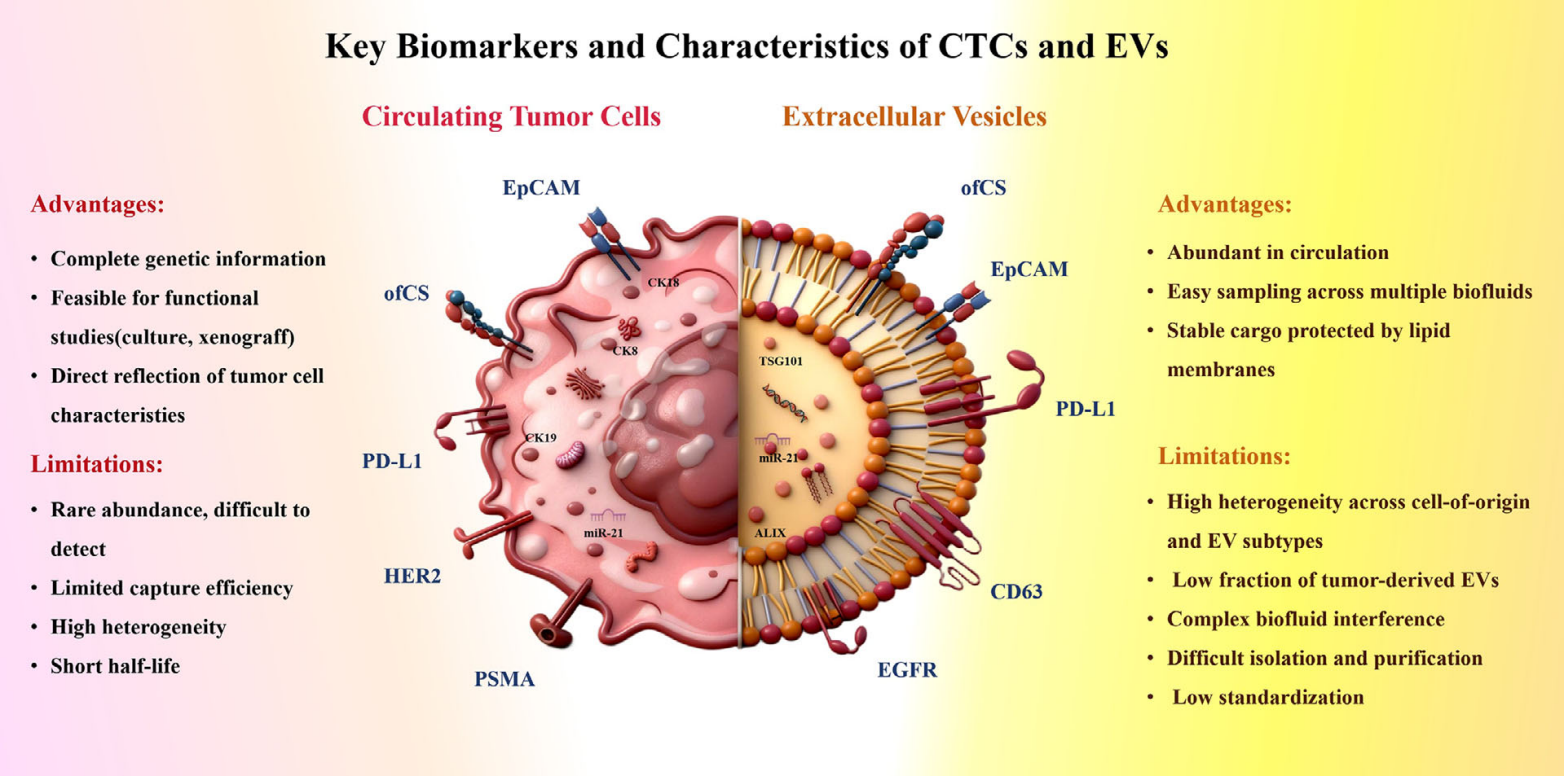
Schematic illustration displaying the biomarkers and detection challenges of CTCs and EVs. The illustration was original work prepared by the authors for this review.

Liquid biopsy has emerged as a key direction in precision medicine research in recent years due to its minimally invasive nature, reproducibility, and dynamic monitoring capabilities. It demonstrates significant potential in early cancer screening, treatment efficacy assessment, and recurrence monitoring. Among various candidate biomarkers, circulating tumor cells (CTCs) and extracellular vesicles (EVs) have garnered significant attention [1]. They serve as complementary tools in liquid biopsy. CTCs retain complete genetic information, which uniquely enables functional studies such as cell culture and xenografts [2]. Meanwhile, EVs benefit from high abundance in circulation and carry stable molecular cargo protected by lipid membranes. Despite these respective advantages, clinical translation for both biomarkers is hindered by common limitations regarding high heterogeneity and difficulties in isolation and purification [3, 4]. However, the specific sources of interference differ fundamentally [5]. For CTCs, the primary obstacles are their rare abundance and short half‐life. This extreme rarity, combined with the overwhelming presence of background leukocytes, limits capture efficiency and reduces sample purity, often masking tumor‐specific signals [6]. In contrast, while total EVs are abundant, tumor‐derived EVs (tEVs) constitute only a small fraction of the overall population. Their analysis is further constrained by complex biofluid interference, as abundant lipoproteins, protein aggregates, and cell debris—many with sizes overlapping those of EVs—complicate their discrimination and reduce detection specificity. Moreover, the small nanoscale size of EVs places additional demands on sensitivity and resolution in downstream assays (Figure 1).

Traditional physical separation methods have limitations in terms of efficiency and purity. While immunological enrichment and molecular recognition strategies can enhance specificity, they often result in marker loss and loss of biological information [7, 8]. In recent years, biomimetic membrane interface engineering has emerged as a potential solution [9, 10]. By constructing biomimetic interfaces composed of natural cell membranes, synthetic lipid, or hybrid membranes, researchers can achieve more efficient molecular recognition while maintaining interface stability [11, 12]. For instance, membranes derived from cancer or immune cells confer strong specific recognition capabilities to platforms [13]; platelet and red blood cell membranes enhance immune evasion and circulation stability [14]; further integration with aptamer or antibody modifications improves target capture efficiency and detection sensitivity [15]. Notably, biomimetic membranes facilitate EV separation and can fuse with vesicle membranes, enabling simultaneous detection of endogenous molecules such as RNAs and membrane proteins [16, 17]. These advancements not only enhance CTC enrichment efficiency and purity but also expand the application potential of EVs in the comprehensive detection of nucleic acids, proteins, and multiple biomarkers.

Existing reviews have either focused separately on CTCs [18, 19] or EVs [20], or examined single platform technologies such as liposomes [21]. Despite the complementary roles of CTCs and EVs in liquid biopsy, no systematic review has examined biomimetic membrane interface engineering approaches for both biomarkers within a unified framework. This gap underscores the necessity for a comprehensive examination of membrane‐based detection strategies for these two critical liquid biopsy targets.

Accordingly, this review integrates CTCs and EVs into a unified biomimetic membranes interface engineering perspective, systematically summarizing their latest advances in liquid biopsy. First, it introduces the sources, preparation, and functionalization strategies of biomimetic membranes, discussing commonly used performance characterization methods. Subsequently, it summarizes the shared biomimetic structural foundations, capture efficiency enhancement mechanisms, and non‐specific binding suppression strategies common to both CTC and EV detection, elucidating the universal advantages of bio‐membrane interfaces as cross‐marker platforms. It then explores their differentiated applications across various detection scenarios, including efficient CTC separation and detection, as well as efficient EV separation, miRNA and protein detection, and synergistic analysis of multiple biomarkers (e.g., miRNA, protein, and mRNA). Finally, this review summarizes key challenges in current research and outlines future development directions. By establishing this cross‐marker review framework, we aim to provide guidance for clinical translation in precision medicine.

## 2 Strategies for Constructing and Modifying Biomimetic Membrane‐Based Interfaces and Carriers

### 2.1 Biomimetic Membrane Sources for Interfacial/Vesicular Engineering

Biomimetic membrane‐based interfacial engineering strategies leverage the fluidity, biomimetic properties, and surface functionalization potential of membrane structures to construct highly efficient platforms for CTC and EV capture/detection. According to the origin of membrane components, these can be primarily classified into synthetic lipids, natural cell membranes, and hybrid cell membranes.

#### 2.1.1 Synthetic Lipid Interfaces/Vesicles

Synthetic membrane interfaces mainly adopt a bottom‐up assembly strategy, utilizing commercially available lipids, polymers, and proteins for construction. The core advantages of selecting such interfaces lie in their highly defined composition, controllable batch‐to‐batch consistency, and ease of standardized production. Furthermore, synthetic membranes can achieve antifouling capabilities, dynamic ligand recognition, target enrichment, and high‐capacity probe loading by mimicking the zwitterionic surfaces, lipid fluidity, and vesicular structures of cell membranes.

In terms of antifouling properties, synthetic membrane interfaces primarily rely on the formation of tightly bound hydration layers. The zwitterionic hydrophilic headgroups of lipid molecules, such as phosphatidylcholine, can induce oriented arrangement of interfacial water molecules through hydrogen bonding, constructing a stable physical barrier. This hydration layer effectively generates steric hindrance, preventing nonspecific binding of blood cells, and other components to the substrate. For example, a cell membrane mimetic surface (CMMS) constructed using phosphorylcholine zwitterionic polymers effectively repelled over 99% of non‐target blood cells by mimicking the electrical neutrality and hydration characteristics of the natural cell membrane outer layer [22]. Similarly, microarray platforms based on supported lipid membranes reduced nonspecific protein adsorption by more than 10‐fold compared to traditional substrates, significantly enhancing the specificity of direct capture of tEVs from complex matrices [23].

Regarding dynamic ligand recognition and target capture, synthetic lipid interfaces demonstrate dual advantages. On one hand, the unique 2D fluid characteristics of lipid interfaces endow surface capture probes with lateral diffusion and dynamic clustering capabilities, significantly enhancing capture efficiency for low‐abundance targets by converting monovalent binding into high‐avidity multivalent binding [24]. On the other hand, charge modulation of synthetic membranes enables rapid target enrichment through electrostatic interactions. For instance, Zhang et al. exploited charge attraction between cationic liposomes and negatively charged EVs to achieve efficient EV anchoring at the interface, and further delivered probes into vesicle interiors through charge‐mediated membrane fusion, enabling in situ detection of intravesicular markers [25, 26].

In probe loading, synthetic membrane interfaces, particularly liposomal nanovesicular structures, exhibit excellent spatial encapsulation and interfacial expandability. Due to the enclosed vesicular structure of lipid bilayers, their interiors can effectively load nanomaterials or probes, improving stability. Meanwhile, the large specific surface area of liposomes provides high‐capacity chemical functionalization interfaces. For example, using magnetic immunoliposomes, researchers can simultaneously modify multiple tumor marker antibodies at high density on a single carrier surface, such as EpCAM, EGFR, and HER‐2, successfully overcoming the false‐negative challenges caused by tumor heterogeneity through multi‐target synergistic strategies [27].

#### 2.1.2 Natural Cell Membrane Interfaces/Vesicles

Natural cell membrane biomimetic construction techniques typically adopt a top‐down strategy, reconstructing and functionalizing natural cell membranes while preserving membrane structural integrity. These membranes can be stably transferred and coated onto nanomaterial surfaces or prepared as biologically active cell membrane vesicle systems for constructing multiscale biomimetic interfaces. The core advantage of such biomimetic systems lies in their ability to maximally inherit the unique membrane protein composition, glycan modification patterns, and mediated dynamic biological functions of source cells, thereby achieving highly mimetic biological camouflage at the functional level and endowing the system with low immunogenicity, effective immune evasion capabilities, and selective recognition and interaction capabilities toward homologous or related biological components.

Red blood cell membranes (RBCM), through natural expression of CD47 and other “don't eat me” signal molecules on their surfaces, can activate the inhibitory receptor SIRPα on macrophage surfaces, effectively reducing clearance efficiency by the mononuclear phagocyte system and significantly prolonging circulation time in vivo or in complex systems [28]. Additionally, the highly hydrated glycocalyx layer covering the RBCM exterior and its uniform negative charge distribution jointly construct a low‐fouling biomimetic interface that significantly inhibits nonspecific adsorption of complex matrix components such as serum proteins, thereby enhancing interface recognition specificity and reliability [29]. Compared to RBCM, white blood cell membranes (WBCM) exhibit advantages in natural trans‐cellular interaction and specific recognition capabilities. WBCM retains abundant adhesion molecules and chemokine receptors that can mimic the endogenous interaction mechanisms between leukocytes and CTCs during metastasis, thereby enhancing capture efficiency. Meanwhile, based on homotypic repulsion properties between leukocytes, WBCM can effectively avoid interference from background leukocytes and improve separation purity [30]. Furthermore, platelet membranes can specifically recognize cancer cell surface receptors through surface integrins such as αIIbβ3 and αvβ3, thereby mediating efficient targeted adhesion [31]. Cancer cell membranes (CCM) demonstrate unique homotypic homing advantages, utilizing surface‐expressed adhesion molecules such as E‐cadherin and Galectin‐3 to achieve high‐affinity capture of homologous cancer cells and tEVs through homotypic interactions [32, 33].

Beyond natural cell membranes, cells can be genetically engineered to stably express specific membrane proteins or ligands, and programmable interfacial membrane vesicles can be obtained through membrane extraction or natural secretion. This strategy can present high‐density, conformationally intact functional proteins on vesicle surfaces, significantly enhancing recognition specificity for CTCs or EVs. Meanwhile, membranes derived from blood cells or immune cells can endow vesicles with low immunogenicity and excellent antifouling performance, reducing nonspecific adsorption. For example, in situ expression of antibody fragments (scFv) on leukocyte membranes can avoid protein activity damage from chemical conjugation and achieve over 100‐fold enhanced CTCs affinity with high‐purity capture [34]. Similarly, another study co‐expressed paramyxovirus receptor‐binding protein hemagglutinin (HA) and fusion protein F on cell membranes to construct genetically engineered vesicles utilizing HN for highly specific recognition of sialic acid receptors on EV surfaces and mediating membrane fusion through F protein, thereby achieving efficient targeted fusion and molecular delivery [35].

#### 2.1.3 Hybrid Membrane Interfaces/Vesicles

Hybrid cell membranes combine membrane components from different sources, integrating their respective unique natural biological advantages. The core advantage of selecting such interfaces lies in their ability to break through functional limitations of single membrane types and achieve enhanced comprehensive performance, including targeted recognition, antifouling properties, and circulation stability through multi‐component synergistic effects.

In dual‐component synergy, hybrid membranes constructed by fusing platelet membranes (PLTM) with WBCM demonstrate excellent synergistic effects. PLTM components significantly enhance capture efficiency by exploiting their natural affinity for tumor cells, while WBCM components substantially reduce nonspecific adhesion to background leukocytes through homotypic repulsion mechanisms, effectively addressing the bottleneck in traditional immunomagnetic beads that struggle to balance high efficiency with high purity [36].

For comprehensive multifunctional optimization, ternary hybrid membranes (RPCM) constructed by further incorporating RBCM, PLTM, and CCM achieve comprehensive functional enhancement. RBCM components significantly improve nanoparticle dispersion stability and long circulation capability in blood; platelet membranes provide immune evasion and tumor homing functions, while CCM achieves EpCAM‐independent capture of heterogeneous CTCs through homotypic adhesion mechanisms. This ternary synergistic strategy achieved over 97% capture efficiency and purity in whole blood, effectively overcoming defects of single‐component membranes such as aggregation or insufficient targeting [31].

Furthermore, to complement the advantages of natural and synthetic membranes, researchers have fused natural EVs, which possess unique tumor homing capabilities but have limited yield, with easily functionalizable synthetic liposomes. This semi‐synthetic hybrid strategy not only preserves the homotypic targeting activity of EVs but also utilizes liposomes to provide excellent antifouling barriers and bioorthogonal reaction sites, thereby successfully addressing the challenge of highly efficient capture of highly heterogeneous melanoma CTCs without relying on specific antibodies [37].

In summary, synthetic lipids, natural cell membranes, and hybrid membranes each possess distinct advantages and limitations. Synthetic lipid membranes exhibit significant advantages in functionalization and loading capacity due to their highly controllable composition and ease of standardized production. However, compared to natural cell membranes, they lack complex biological functions such as natural immune evasion signals like CD47 or tumor homing receptors, and the physical stability of lipid bilayers is relatively weak. They are prone to fusion or leakage in complex physiological environments, which limits their application stability during in vivo circulation or prolonged operations. Natural cell membranes can maximally preserve the biological functions of source cells, demonstrating superior performance, particularly in immune evasion and targeted recognition. Nevertheless, they face challenges, including limited membrane sources, relatively complex preparation procedures, susceptibility of membrane protein activity to damage during extraction, and difficulty in controlling batch‐to‐batch variations, which to some extent restrict their large‐scale standardized applications. Hybrid membranes enhance interface multifunctionality and targeting performance by combining characteristics of different membranes, but their construction process is relatively complex, involving extraction and fusion procedures of multiple cell membranes. How to precisely control the proportion of different membrane components during the fusion process and ensure long‐term stability of fused membranes remain challenges for achieving large‐scale standardized production.

### 2.2 Construction Strategies of Biomimetic Membrane‐Based Carriers

**FIGURE 2:**
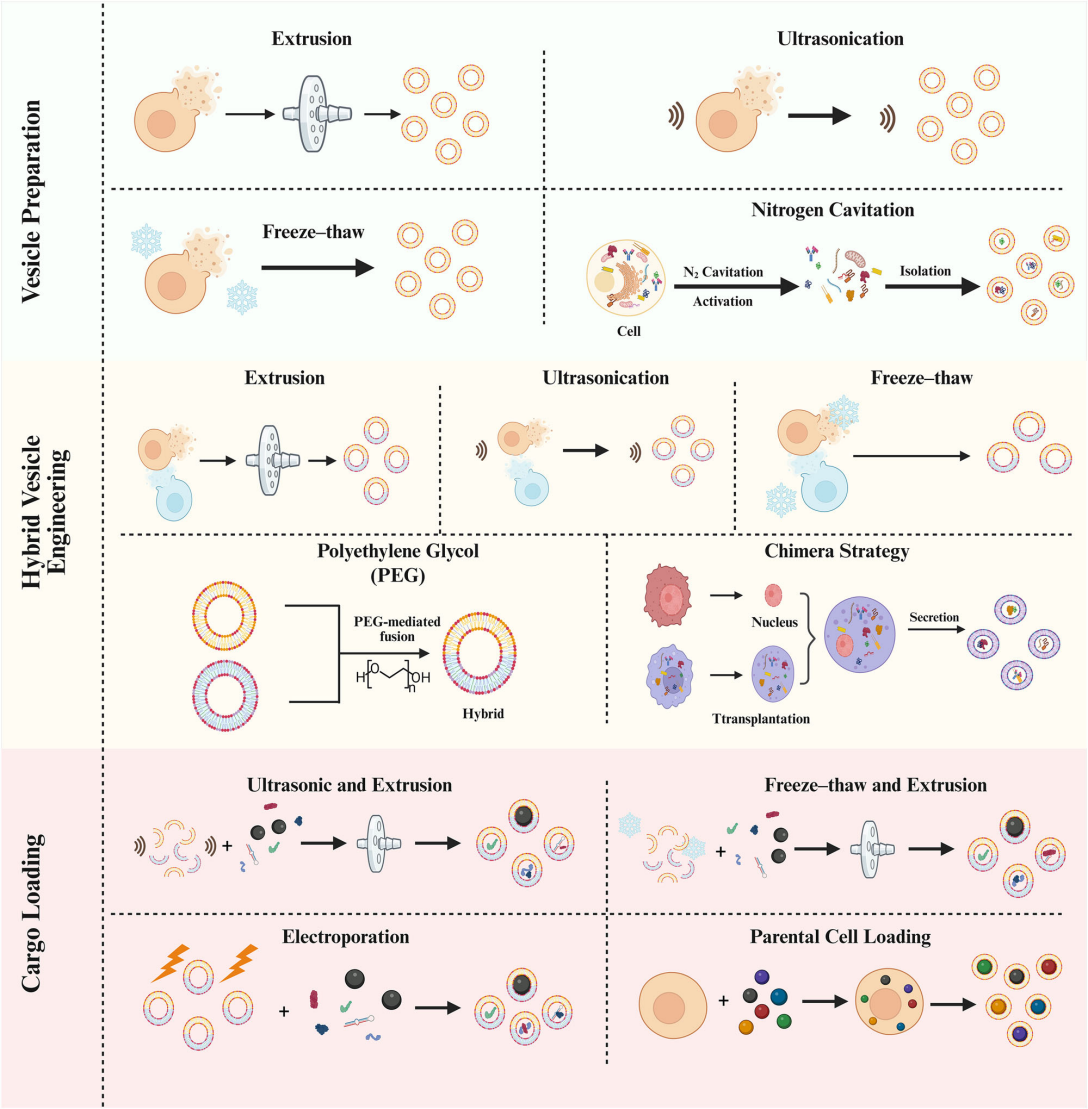
Schematic illustration of biomimetic membrane vesicle preparation, hybrid vesicle engineering, and cargo loading strategies. The illustration was generated using BioRender under a licensed subscription (Agreement number: DI295PEMHQ).

The construction of biomimetic membrane‐based carriers involves three key steps: preparation of membrane vesicles, formation of hybrid vesicles, and loading of functional cargos. Various physical and chemical methods have been developed for each step, offering different advantages in terms of efficiency and preservation of membrane functionality (Figure 2).

#### 2.2.1 Preparation Methods of Biomimetic Membrane Vesicles

Taking cell membrane vesicles as an example, commonly used preparation methods include extrusion, ultrasonication, freeze‐thaw cycling, and nitrogen cavitation, each possessing distinct characteristics and applicable scenarios.

Extrusion is the most commonly employed preparation method. Through hypotonic lysis, mechanical membrane disruption, and differential centrifugation, intracellular contents are removed, and cell membranes are collected. The collected membranes are repeatedly passed through polycarbonate membranes for physical extrusion, thereby forming nanoscale membrane vesicles [38]. The advantage of this method lies in its ability to precisely control vesicle size and achieve high uniformity, although the operation is relatively cumbersome.

Nitrogen cavitation can enhance vesicle yield while maintaining membrane protein activity. Cell suspensions are filled into a nitrogen cavitation chamber, where pressure of 400 to 500 psi is applied under low temperature conditions, followed by rapid decompression to achieve cell disruption. Membrane vesicles can be obtained by centrifugal collection of the precipitate [39]. This method offers high yield and better preserves the native conformation and function of membrane proteins, but requires specialized equipment with relatively high costs.

Ultrasonication disrupts cell membranes through ultrasonic treatment and reorganizes them into nanoscale monolayer vesicles. Due to the randomness of disruption, the resulting vesicles may present either forward or reverse membrane orientations and may contain membrane components from organelles such as endoplasmic reticulum and Golgi apparatus [40]. This method is simple and rapid to operate, but may lead to membrane protein denaturation and exhibits a broader vesicle size distribution.

Freeze‐thaw cycling involves subjecting membrane suspensions to repeated freezing and thawing cycles. Ice crystal formation and osmotic pressure changes during freezing cause membrane structure rupture, while membrane fragments spontaneously reorganize into smaller vesicles during thawing [41]. This method is gentle and cost‐effective, suitable for large‐scale preparation, but has lower precision in vesicle size control and may require a combination with other methods for further optimization.

#### 2.2.2 Methods for Preparing Hybrid Vesicles

Unlike naturally secreted EVs from cells, hybrid vesicles are constructed through artificial methods that fuse membrane structures from different sources. Main methods include co‐extrusion, ultrasonication, freeze‐thaw cycling, polyethylene glycol (PEG)‐induced fusion, and the chimera method.

Co‐extrusion is the most commonly used method for preparing hybrid vesicles, typically combined with ultrasonication treatment. Li et al. fused EVs derived from bone marrow mesenchymal stem cells (MSCs) with platelet membranes through ultrasonication and extrusion techniques. Transmission electron microscopy (TEM) results showed that the extrusion process did not disrupt vesicle structure, with hybrid vesicles displaying no significant difference in particle size compared to original EVs and exhibiting uniform size distribution [42]. Rayamajhi et al. similarly employed ultrasonication combined with extrusion to hybridize small EVs (sEVs) derived from mouse macrophages with synthetic liposomes, with the size uniformity of hybrid vesicles significantly superior to that of sEVs alone [43]. The advantage of this method lies in controllable operation and intact vesicle structure.

Freeze‐thaw cycling induces membrane fusion through repeated freezing and thawing cycles. Lipid membrane dehydration, phase transition, and lateral phase separation during freezing promote membrane restructuring and fusion. Sato et al. employed this method to fuse exosome membranes with liposomes, constructing hybrid exosomes [44]. This method is operationally simple and does not require chemical reagents, thereby avoiding potential contamination from substances such as calcium ions or PEG. However, the freeze‐thaw conditions need to be optimized to balance fusion efficiency and membrane integrity.

PEG‐induced fusion utilizes polyethylene glycol as a chemical inducer to promote membrane fusion, with advantages of simple operation and high fusion efficiency. Piffoux et al. mediated the fusion of EVs with liposomes using PEG 8000, with cryo‐electron microscopy confirming the occurrence of complete fusion. This method can be combined with co‐extrusion to achieve higher fusion rates and a more ideal size distribution [45]. However, attention should be paid to potential membrane contamination from PEG residues, which should be sufficiently removed during application.

The chimera method constructs chimeric cells through nuclear transfer technology, enabling them to naturally secrete hybrid vesicles possessing dual cellular characteristics. Wang et al. isolated cell nuclei from various tumor cells (E.G7 lymphoma, 4T1 breast cancer, B16 melanoma, etc.) and introduced them into enucleated M1‐type macrophages to construct chimeric cells. These chimeric cells could naturally secrete chimeric nanovesicles bearing both tumor and macrophage membrane characteristics [46]. This method can obtain hybrid vesicles with stronger functionality, but the preparation process is complex, requiring advanced technical capabilities such as nuclear transfer.

#### 2.2.3 Methods for Loading Functional Probes or Encapsulating Nanomaterials inside Biomimetic Membranes

Common methods for loading functional probes or nanomaterials into biomimetic membrane vesicles include ultrasonication‐extrusion, freeze‐thaw cycling‐extrusion method [47], electroporation [48], and parent cell loading [49]. A comprehensive quantitative analysis of these methods, evaluating their respective advantages and limitations in terms of cost, scalability, and product quality, is summarized in Table 1.

**TABLE 1:** Comparative analysis of common methods for preparing biological membrane interfaces.

The type of preparation method	Scalability	Membrane protein integrity	Batch‐to‐batch reproducibility	Cost	Time	Advantages	Disadvantages	References
**Extrusion**	Scalable from mL to ∼10^4^ mL with pressurized extrusion, but repeated 7–20‐cycle processing still limits throughput	High integrity, uniform, and stable structure, with no significant protein damage	Extremely high. Strictly limited by the filter pore size, ensuring a highly consistent size distribution across batches	Moderate. Entry‐level manual sets are affordable (∼$1250), while automated systems are costlier (∼$4k–$11k); consumable costs are relatively low *Sources*: *Avanti Polar Lipids (Mini‐Extruder); Genizer LLC (HandExtruder); ATS Engineering Limited*	Time‐consuming and labor‐intensive. A multi‐step process requiring hydration and repeated mechanical cycling (10–20 passes); manual operation is slow and limits throughput	Excellent size uniformity (low PDI) and reproducibility; free from chemical residues or metal contamination	Labor‐intensive and time‐consuming; filters clogeasily with concentrated samples; relatively low encapsulation efficiency	[50, 170, 171, 172, 173, 174]
**Ultrasonication**	Supports large batch volumes, but more suited for laboratory scales	High energy risks can disrupt membrane structure and function, leading to membrane instability and potential protein denaturation	Low. Highly sensitive to operational details (e.g., probe position, temperature), leading to high variability and difficulty in standardization	Low. Equipment is accessible with a wide price range (∼$780‐$6100); negligible consumable costs beyond standard vessels *Sources*: *Shanghai Lichen (JY96‐IIN); Ningbo Scientz; U.S. Solid; Drawell Scientific; Qsonica LLC (Q700)*	Rapid. The fastest method (typically minutes); requires intermittent cooling pauses, but offers the highest overall time efficiency for small batches	Simple, rapid, and accessible; ideal for producing small vesicles	High energy risks, lipid oxidation, and protein damage; Probe tips can introduce metal particle contamination; Broad size distribution (poor uniformity); Often used in combination with extrusion to improve size uniformity	[175, 176, 177, 178]
**Freeze‐thaw Cycling**	Suitable for large‐scale preparation, but may require combination with extrusion for further optimization of vesicle size and efficiency	Relatively gentle and preserves membrane protein function	Variability in freezing‐thawing cycles and ice crystal formation can affect reproducibility across batches	Cost‐effective and does not require expensive equipment or chemicals	Requires multiple freezing and thawing cycles, making it a time‐consuming process	Simple and cost‐effective; Gentle on membrane integrity; Suitable for large‐scale preparation·	Lower precision in vesicle size control; May require combination with extrusion for optimal results; Time‐consuming process	[55]
**Electroporation/ Microfluidic electroporation**	Limited by batch processing, difficult to scale up for large volumes Microfluidic electroporation enables continuous‐flow processing with ∼100× higher throughput than cuvette‐based electroporation. Large‐scale demands are met via expanded active areas or multi‐layer stacking designs	Excessive parameters cause irreversible rupture. Can disrupt membrane integrity, potentially affecting protein activity. May also cause lipid peroxidation and cargo aggregation	Reproducibility may vary across batches due to differences in voltage, timing, and sample conditions	High. Requires a complex system (generator + pump, ∼$13k‐$30k) and high‐cost disposable microfluidic chips (∼$275–$690 each) *Sources*: *Harvard Bioscience (ECM 399, Gemini X2, Pump 11 Pico Plus); Bio‐Rad Laboratories (Gene Pulser Xcell); KD Scientific (Legato 100); Micronit Microtechnologies (Chips)*	Quick at small scales, but requires longer optimization when scaling up	High encapsulation efficiency; Microfluidic electroporation enables continuous flow production; solvent‐free	Accompanying Joule heating, dramatic pH changes, and electrochemical byproducts	[179]
**Parent cell loading**	Slow loading cycles, and suffer from batch heterogeneity, difficult to scale up for large volumes	High. Preserves natural membrane structure and function, as vesicles are naturally secreted from cells	Medium. Variability in nanoparticle uptake and cell type may affect consistency across batches	Relatively low cost since it is free of specialized equipment, though dependent on cell culture and nanoparticle material	Long. Requires extended incubation times for nanoparticle uptake and vesicle release	Maintains natural membrane integrity and protein functionality; Can load a variety of nanoparticles, including inorganic and organic types	Lower loading efficiency compared to other methods; Limited to nanomaterials that can be internalized by cells; Time‐consuming and not suitable for high‐throughput applications	[49, 61]

The combined ultrasonication‐extrusion method is the most commonly used strategy for coating nanomaterials. In a classic proof‐of‐concept study, researchers first obtained membrane skeletons through hypotonic lysis of red blood cells, then restructured the membranes into vesicles through ultrasonication treatment and mechanical extrusion through porous membranes. When these membrane vesicles were co‐extruded with pre‐prepared poly(lactic‐co‐glycolic acid) nanoparticle cores, transmission electron microscopy revealed that the resulting nanoparticles exhibited a typical core‐shell structure, with the membrane layer completely coating the periphery of the core [50, 51]. Kang et al. employed the same technique to coat nanoparticle surfaces with neutrophil membranes [52], while Wu et al. achieved coating with melanoma cell membranes [53]. This method offers high coating efficiency and good reproducibility, but the ultrasonication process may affect the structure and function of membrane proteins [47, 54].

Some studies have combined freeze‐thaw and extrusion methods for nanomaterial coating. This involves using freeze‐thaw cycles to fuse cell membrane fragments with exogenous phospholipids into hybrid vesicles, followed by repeated extrusion through polycarbonate membranes to achieve uniform size, and finally co‐extruding these vesicles with nanoparticles [55]. While this approach leverages both membrane fusion and size control advantages, multiple extrusion cycles are still required for optimal uniformity.

Electroporation forms nanoscale pores in membranes through transient high‐voltage pulses, allowing substances to enter vesicle interiors. This method has been successfully applied to fluorescent probes [56, 57], and magnetic nanoparticles [58]. Although electroporation demonstrates high efficiency, its accompanying Joule heating, dramatic pH changes, and electrochemical byproducts (such as ROS and metal ions) not only may disrupt membrane integrity and protein activity but also readily induce lipid peroxidation and spurious aggregation of nucleic acid cargo [59, 60]. Microfluidic and nanoelectroporation technologies can achieve gentler and more efficient loading through miniaturized designs [59, 60]. For example, Rao et al. developed a microfluidic system integrating rapid mixing with electroporation, successfully achieving efficient coating of magnetic nanoparticles with red blood cell membranes [58]. However, high costs, complex processes, and scale‐up bottlenecks still limit its widespread application.

The parent cell loading method involves co‐incubating cells with nanomaterials (such as iron oxide nanoparticles, gold nanoparticles, or quantum dots), utilizing cellular endocytosis and secretion mechanisms so that cells naturally secrete vesicles containing nanomaterials during culture [61]. Silva et al. demonstrated that this method can be applied to encapsulate inorganic nanoparticles of different shapes, sizes, chemical compositions, and surface modifications [49]. This method maintains the natural structure and function of membranes, but has lower loading efficiency, longer cycles, and is only suitable for nanomaterials that can be taken up by cells.

### 2.3 Functional Modification of Biomimetic Membranes

**FIGURE 3:**
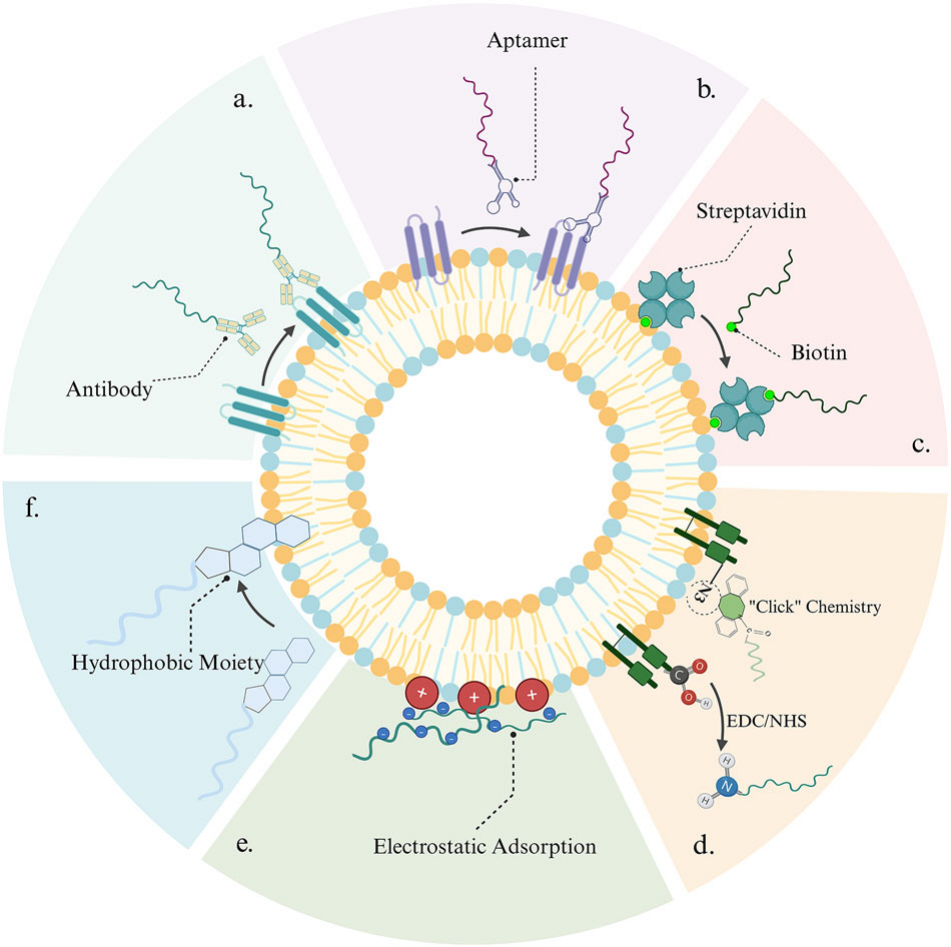
Strategies for ligand immobilization on biomimetic membrane interfaces. The illustration was generated using BioRender under a licensed subscription (Agreement number: PV295PFGOQ).

The antibody‐mediated surface modification technique capitalizes on the highly specific and robust affinity binding between the target antigen and its corresponding antibody. Precise molecular immobilization at the biointerface is accomplished by coupling oligonucleotides or alternative functional probes to the antibody, enabling targeted binding to specific surface epitopes on the vesicles (Figure 3a). A significant advantage is the antibody's exceptional molecular recognition capacity, which facilitates the selective labeling of distinct EV subsets, even within highly heterogeneous matrices. However, this methodology is constrained by several factors. The acquisition of high‐quality antibodies is costly and mandates rigorous quality assurance. Moreover, the critical antibody‐oligonucleotide conjugation process is technically demanding, requiring considerable experimental proficiency. Critically, the intrinsically high molecular weight and potential batch‐to‐batch variability of the antibodies themselves can detrimentally impact the overall reproducibility and stability of the platform [62].

Aptamers are nucleic acid ligands capable of folding into specific 3D structures to recognize target molecules with high affinity. The principle of aptamer‐based surface modification relies on their specific binding to particular epitopes on the vesicle surface, thereby anchoring functional molecules connected to their termini onto the vesicle (Figure 3b). Compared to antibodies, aptamers offer significant advantages, including a low molecular weight, ease of chemical synthesis and modification, lower cost, and high batch‐to‐batch consistency. However, their 3D conformation is susceptible to environmental factors such as ionic strength and temperature, which can compromise both binding stability and affinity [63]. Concurrently, the screening of high‐affinity, low off‐target aptamers remains a time‐consuming process requiring extensive optimization rounds. Nonetheless, due to their exceptional specificity and programmability, aptamers remain one of the key molecular tools for achieving precise surface engineering of vesicles.

The biotin‐streptavidin system is recognized as an exceptionally stable immobilization method, often likened to a covalent linkage, due to its ultrahigh affinity (Kd ∼10^−^^1^ ^5^ M). The modification principle typically involves a two‐step process: first, a biotinylated molecule is anchored onto the surface of the vesicle membrane. Subsequently, the tetravalent binding sites of streptavidin are utilized to construct a robust bridging layer, which then allows for the introduction of functional elements such as biotinylated antibodies, achieving high‐density, directional immobilization of ligands on the vesicle surface (Figure 3c) [32, 64]. This system offers not only extreme binding strength but also the advantage of operation under mild conditions, which maximally preserves the biological activity of the ligands and provides high versatility for various molecular types [24, 65]. Nonetheless, this approach is limited by the challenge of effective elution, which may interfere with subsequent downstream analyses.

Covalent modification, by forming stable chemical bonds between functional molecules and the vesicular membrane components, achieves durable and dissociation‐resistant surface functionalization, making it one of the most classic and robust interface engineering strategies available (Figure 3d) [66]. Specifically, the 1‐ethyl‐3‐(3‐dimethylaminopropyl)carbodiimide (EDC) and *N*‐hydroxysuccinimide (NHS) system activates carboxyl groups on the membrane surface to form amide bonds with amino groups, enabling stable conjugation of antibodies or other ligands. However, the random distribution of lysine residues leads to uncontrolled modification sites, which can result in ligand disorientation and compromise biological activity [67]. In contrast, maleimide–thiol chemistry utilizes the sulfhydryl group of cysteine to undergo a thia‐Michael addition, offering significantly higher site specificity, thereby markedly enhancing both coupling efficiency and uniformity, although excessive thiol modification may potentially perturb the lipid bilayer structure [67, 68]. More recently, click chemistry has emerged as a crucial tool for precise modification due to its reliance on the bio‐orthogonal cycloaddition between azide and alkyne groups. This reaction rapidly and specifically generates a stable triazole ring structure under mild conditions, offering both high coupling efficiency and excellent biocompatibility [37]. Overall, while covalent modification methods are reliable and stable, a careful balance must be struck among site‐controllability, preservation of membrane integrity, and operational complexity.

Electrostatic adsorption represents a class of non‐covalent surface modification strategies founded on electrostatic interactions and van der Waals forces. The mechanism leverages the electrostatic attraction between positively charged lipids on the membrane surface and negatively charged ligands, such as nucleic acid aptamers, enabling the spontaneous adsorption and interface assembly of functional molecules (Figure 3e). Relative to covalent coupling, this methodology eliminates the need for supplementary chemical reactions or complex linkers, featuring mild operational conditions that maximally preserve the ligand's native conformation and biological activity. Furthermore, it offers advantages such as low cost and rapid construction [69]. However, because electrostatic adsorption is fundamentally a weak non‐covalent interaction, its stability is susceptible to degradation by environmental factors, including ionic strength, pH, and serum proteins. Additionally, the distribution of ligands on the membrane surface is often random, making it challenging to achieve controlled orientation and uniformity.

In addition to the aforementioned techniques, hydrophobic anchoring is a widely employed strategy for modifying the surface of vesicles. Its mechanism involves conjugating the desired ligand with a hydrophobic moiety, such as cholesterol or a lipid, enabling the conjugate to spontaneously insert into the core of the phospholipid bilayer via hydrophobic interactions (Figure 3f). This approach obviates the need for complex chemical coupling reactions, is straightforward to execute, and exhibits high applicability, leading to its widespread use in biomimetic membrane surface engineering [27, 36, 70]. However, hydrophobic insertion presents certain limitations: high‐density hydrophobic molecules may perturb the membrane structure and potentially affect vesicular homeostasis. Furthermore, amphiphilic oligonucleotides might spontaneously form nano‐aggregates rather than achieving effective insertion. Moreover, this strategy inherently lacks precise control over the insertion site and density, which can impact experimental reproducibility and the accuracy of subsequent quantitative analyses.

### 2.4 Performance Characterization of Biomimetic Membranes Interface Functionalization

In research on functionalized biomimetic membrane interfaces, the resulting interfaces must undergo systematic characterization to ensure modification efficiency, structural integrity, and biological function, thereby supporting the reliability of subsequent applications.

Quantitative modification analysis represents the primary step in performance evaluation. Determination of total protein concentration at the membrane interface using bicinchoninic acid (BCA) or Bradford assays enables protein content quantification and provides a baseline for modification efficiency calculations. For precise determination of surface antibody or nucleic acid grafting density, conjugation efficiency can be calculated by directly measuring conjugated antibody/nucleic acid content or indirectly measuring free antibody/nucleic acid in the centrifugation supernatant [71, 72, 73]. Fluorescence methods can also quantify surface antibody or nucleic acid density [74].

**FIGURE 4:**
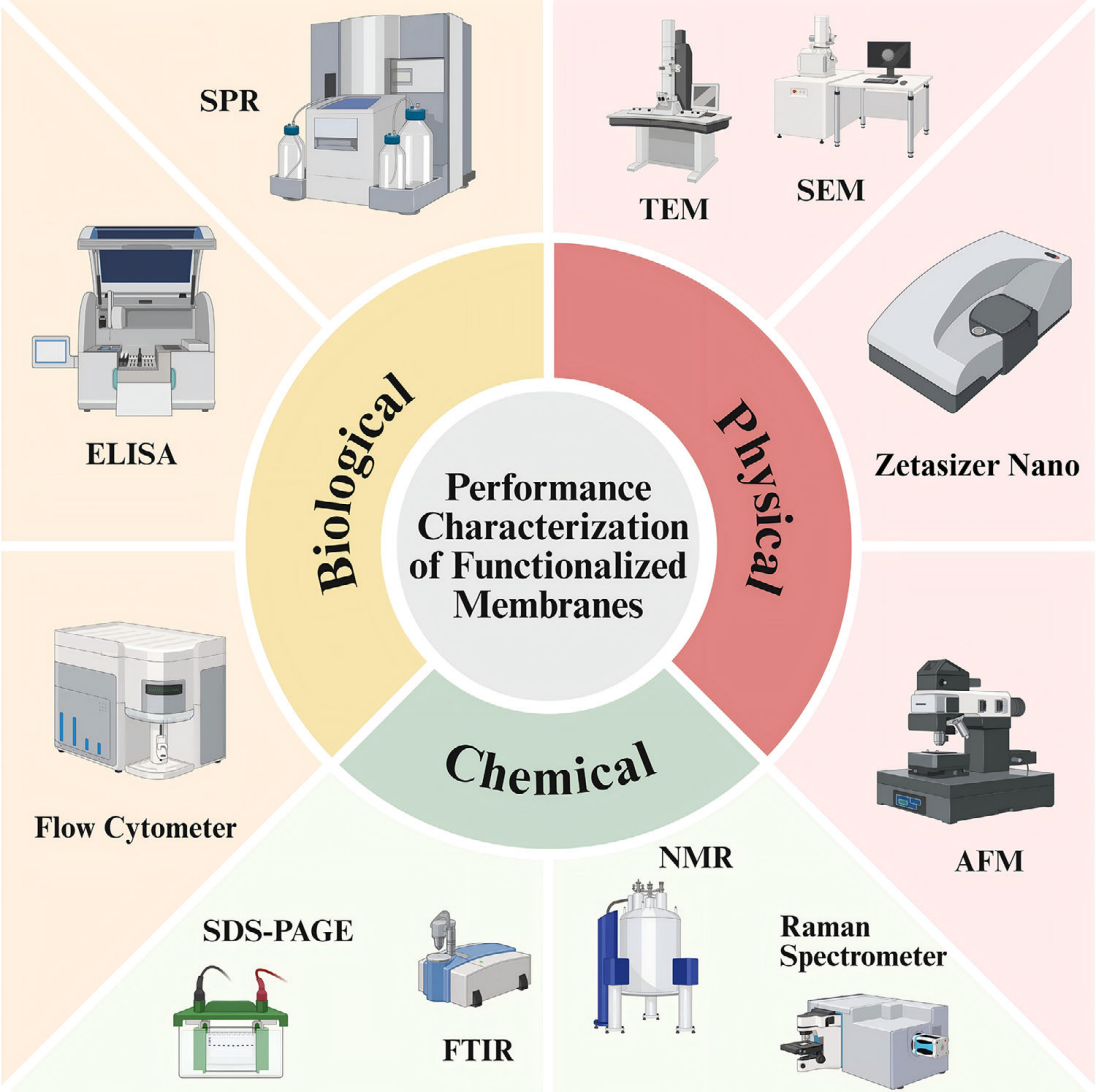
Performance characterization of biomimetic membranes interface functionalization. The illustration was generated using BioRender under a licensed subscription (Agreement number: SH295PFOMS).

Multidimensional physicochemical characterization is crucial for ensuring membrane interface quality (Figure 4). Regarding physical characterization, for nanoscale membrane structures such as liposomes and vesicles, particle size distribution and surface charge (Zeta potential) are typically assessed through dynamic light scattering (DLS) and Zeta potential analyzers, which reflect colloidal stability and homogeneity following modification [75, 76]. Morphological integrity requires visual confirmation through TEM, scanning electron microscopy (SEM), or atomic force microscopy (AFM) [77, 78]. Regarding chemical characterization, successful conjugation of ligands to membrane proteins can be preliminarily assessed through molecular weight migration in dodecyl sulfate polyacrylamide gel electrophoresis (SDS‐PAGE) [79, 80]. For antibody‐modified interfaces via maleimide chemical conjugation, SDS‐PAGE analysis under reducing and non‐reducing conditions can further confirm thioether bond formation and the structural integrity of antibody heavy and light chains [71]. Specific chemical bond formation and molecular structural changes require confirmation through molecular spectroscopy techniques, including Fourier‐transform infrared spectroscopy (FTIR), nuclear magnetic resonance (NMR), or Raman spectroscopy [81]. It should be noted that the conjugation process may disrupt antibody or nucleic acid structure, leading to reduced or altered biological activity. To ensure biological activity retention, evaluating integrity following nanoparticle binding is essential. Enzyme‐linked immunosorbent assay (ELISA) confirms immunoreactivity by detecting specific binding of functionalized interfaces to immobilized target antigens [82, 83]. Flow cytometry confirms receptor preservation, integrity, and correct orientation following membrane isolation and nanoparticle coating [84]. Surface plasmon resonance (SPR) provides binding kinetic parameters, including dissociation constant Kd, association rate kon, and dissociation rate koff, enabling quantitative assessment of ligand‐target interaction affinity [85].

## 3 The Unified Platform of Biomimetic Membranes Technology in CTC and EV Detection

Biomimetic membranes technology provides a unified platform for detecting CTCs and EVs. The core of this technology lies in mimicking natural membrane structures and interfacial environments, enabling flexible recognition and efficient capture of both types of biomarkers. Compared to traditional detection methods that rely on rigid solid‐phase materials, Biomimetic membranes incorporate biomimetic principles to balance detection sensitivity, target integrity, and feasibility for downstream analysis.

### 3.1 Mechanisms for Enhanced Capture Efficiency

The key mechanism by which biomimetic membrane interfaces significantly enhance capture efficiency lies in the dynamic multivalent binding effect mediated by lateral fluidity. Unlike static receptor fixation on rigid substrates, biomimetic membranes allow anchored receptor molecules (such as antibodies or aptamers) to maintain lateral mobility on the membrane surface. Once the target contacts the interface, receptors can rapidly cluster toward binding sites, forming high‐density spatial multivalent recognition clusters with tumor cell surface antigens or vesicle membrane proteins. Quantitative studies demonstrate that this fluid biomimetic nanointerface with active recruitment capability produces significant synergistic effects, with affinity enhancement up to 10^4^‐fold and approximately 7‐fold improvement in capture efficiency in blood compared to monovalent aptamer‐functionalized chips [86]. Beyond this dynamic fluidity, different biomimetic membranes inherently possess cell‐type‐specific surface proteins that enable natural targeting capabilities. These include chemokine receptors and adhesion molecules on neutrophil membranes [30], integrins on platelet membranes [31], and homotypic recognition markers on cancer cell membranes, providing multimodal recognition mechanisms that single chemically conjugated ligands cannot replicate [31, 33]. Additionally, the inherent softness of lipid bilayers provides important biomechanical protection in high‐flow environments, effectively buffering collision stress between target cells and the interface and reducing mechanical damage risk. This is crucial for maintaining CTC viability and integrity and ensuring downstream single‐cell analysis quality.

Compared to biomimetic membrane interfaces, nanostructures such as nanopillars and spherical nanoparticles have long been applied to enhance CTC and EV capture efficiency. Their advantages primarily manifest in physical enhancement effects at the interface geometry and mass transfer levels. By constructing 3D nanotopological structures, these platforms significantly increase effective specific surface area and enhance the contact frequency and residence time between cells or vesicles and functionalized interfaces, thereby improving capture efficiency. Furthermore, nanoscale structures match the size of a cell or vesicle, facilitating multipoint contact and enhanced adhesion stability. For example, integrating nanostructured silicon substrates with microfluidic chaotic micromixers can enhance near‐wall mass transfer to improve CTC capture efficiency [87]. High‐density functionalized interfaces using ZnO nanowire arrays can significantly enhance capture capability for CD9‐positive exosomes while enabling controlled release [88]. However, it is noteworthy that recognition molecules in these nanostructured platforms are typically still fixed to substrate surfaces in static and rigid manners, with their capture performance primarily depending on increased ligand density and enhanced geometric contact.

Introducing biomimetic membrane interfaces into nanomaterial or micro‐nanostructured systems can achieve mechanistic complementarity and synergistic enhancement. In these systems, nanomagnetic beads and micro‐nanostructures improve contact frequency between targets and interfaces by providing high specific surface area and enhanced fluid mixing effects, while biomimetic membrane interfaces enable efficient and gentle target capture through dynamic multivalent clustering, natural targeting capability, and biomechanical protection. Compared to traditional strategies that statically fix ligands on nanomagnetic beads through covalent bonds or biotin‐streptavidin systems, biomimetic membrane‐modified nanomagnetic beads can actively rearrange recognition molecules during binding processes, thereby significantly alleviating molecular crowding and steric hindrance limitations. Rao et al. systematically compared bare magnetic beads surface‐coupled with the same density of anti‐EpCAM antibodies versus platelet‐leukocyte hybrid membrane‐modified magnetic beads. Results showed that the latter nearly doubled CTC capture efficiency, and this advantage did not stem from increased ligand density but rather from membrane fluidity‐mediated multivalent binding enhancement [36]. Similar synergistic effects are even more evident in microfluidic chips. Nanopillar arrays in deterministic lateral displacement (DLD) chips achieve CTC sorting through size effects while significantly increasing interface area and altering local flow fields to promote target‐interface contact. However, frequent cell collisions with rigid micropillars under high‐flow conditions easily cause mechanical damage. Shen et al. constructed an antibody‐engineered red blood cell membrane interface on the triangular nanopillar surfaces of DLD chips, demonstrating that this strategy maintains the sorting advantages of nanostructures while introducing three biomimetic membrane benefits. First, membrane fluidity promotes dynamic clustering of recognition molecules. Second, binding affinity is significantly enhanced, with dissociation constants reduced 13.3‐fold compared to rigid functionalized systems. Third, membrane softness effectively buffers high‐flow collision stress, thereby significantly improving capture efficiency while protecting CTC integrity [89]. Similar mechanisms hold true for EV capture. The FluidmagFace‐Chip system introduces supported lipid bilayer‐modified affinity magnetic beads into herringbone microfluidic chips, achieving synergistic effects of microfluidic‐enhanced mass transfer, magnetic response enrichment, and membrane fluidity multivalent recognition. Its EV capture efficiency significantly exceeds traditional static functionalized systems using the same number of magnetic beads [90].

In summary, nanostructured platforms and biomimetic membrane interfaces share commonalities but also possess fundamental differences in improving CTC and EV capture efficiency. Both enhance capture performance by increasing target‐interface contact probability, but nanostructures primarily provide passive enhancement at the geometric and mass transfer levels, whereas biomimetic membrane interfaces functionally complement through fluidity‐mediated active molecular rearrangement, natural targeting recognition, and biomechanical buffering. When combined, nanomaterials and micro‐nanostructures provide a high specific surface area to address the question of “how to achieve more contact”, while biomimetic membrane interfaces achieve dynamic recognition and biomechanical protection to address “how to bind more strongly, stably, and gently”. The two produce synergistic enhancement, providing innovative strategies for liquid biopsy technology.

### 3.2 Inhibition and Control of Nonspecific Binding

In complex matrices such as blood, non‐specific adsorption of plasma proteins and non‐specific adhesion of non‐target cells can severely interfere with detection results. The Biomimetic membranes effectively suppresses background noise through multiple mechanisms. First, glycolipids and glycoproteins on the membrane surface can form a hydration layer resembling a glycocalyx, effectively reducing plasma protein adsorption [91]; combined with PEG or zwitterionic polymer modifications, this further enhances anti‐contamination properties. Second, by regulating surface charge density and hydration layer thickness, a balance can be achieved between repelling non‐target molecules and maintaining specific binding. Particularly when constructing biomimetic interfaces using membranes derived from blood cells, leukocyte membranes naturally reduce immune‐related non‐specific binding due to their innate immune regulation and self‐recognition properties; while erythrocyte membranes express the “do not eat me” signal via surface CD47 protein, significantly reducing adhesion of plasma proteins and non‐target particles. Thus, blood cell‐derived biomimetic membranes exhibit effective anti‐non‐specific adsorption properties in complex biofluids, substantially improving detection signal‐to‐noise ratios. This property is particularly crucial for EV detection: due to EVs' nanoscale size and diverse surface markers, achieving highly specific separation from serum protein backgrounds poses significant challenges [92]. By mimicking natural membrane environments, bio‐membrane interface technology not only facilitates the identification and capture of EVs from diverse sources but also enables their cellular origin tracing, thereby enhancing clinical application value [93].

Moreover, although biomimetic membranes exhibit excellent biocompatibility, nonspecific adsorption remains unavoidable in complex physiological environments. The core bottleneck lies in the formation of a protein corona on the surface of biomimetic membranes after contact with bodily fluids; this structure masks the original functionalized sites and triggers nonspecific interactions [94, 95]. To address this issue, strategies such as microscale regulation of the physicochemical properties of biomimetic membranes (e.g., charge neutralization and size optimization), as well as engineered pre‐coating approaches including artificial protein coronas, are expected to interfere with protein adsorption kinetics and thereby reduce nonspecific adsorption [96, 97, 98, 99].

## 4 Differentiated Applications of Biomimetic Membranes in CTC and EV Detection

### 4.1 Detection of CTCs Based on Biomimetic Membranes

This section reviews recent advances in CTC enrichment technologies based on biomimetic membrane interfaces, covering three main strategies, namely CTC detection based solely on biomimetic membranes, CTC enrichment combining biomimetic membrane interfaces with magnetic beads, and microfluidic CTC enrichment integrating nanomaterials and biomimetic membranes. The key advantages and main parameters of these methods are systematically summarized in Table 2.

**TABLE 2:** Representative strategies and applications of biomimetic membrane in CTC detection.

Strategy categories	Biomimetic membrane Source	Functionalized ligands	Key advantages	Performance metrics (Capture Rate/Purity, etc.)	Clinical sample testing	References
Detection of CTCs based solely on the biomimetic membrane	Supportive lipid bilayer	anti‐EpCAM antibody	Lateral mobility of lipid bilayer enables dynamic anti‐EpCAM clustering for enhanced CTCs binding affinity with preserved cell viability and effective blood cell resistance	Capture efficiency: >97%, Capture Purity: >95%	Only simulated samples were used.	[24]
	Cell membrane mimetic surface	FA and RGD peptide	Biomimetic zwitterionic interface with excellent anti‐biofouling properties combined with dual‐ligand modification enables cost‐effective and highly selective CTC capture	Capture efficiency: 91%; Capture purity: 89%	Only simulated samples were used.	[22]
	Red blood cell membrane	Anti‐EpCAM antibody and FA	Synergistic integration of RBCM anti‐fouling properties and C_3_N_4_‐based self‐calibration achieves ultrasensitive CTC detection in complex serum with prolonged sensor lifetime	LOD: 3 CTCs / mL	Only MCF‐7 cells in buffer	[100]
	Tumor cell membrane	Cell membranes derived from MCF‐7 cells	Integration of AuNPs@Mem‐based signal enhancement and erythrocyte membrane anti‐fouling properties achieves highly sensitive SPR detection of CTCs via tumor membrane protein‐specific recognition	LOD: 868.11 particles/mL	Only simulated samples were used.	[101]
	Red blood cell vesicle	FA	Simple fabrication of autologous erythrocyte vesicles with inherent immune evasion and minimal toxicity for integrated in vitro CTC capture and in vivo tumor imaging via dual‐functional targeting and fluorescence capabilities	Capture efficiency: >95%; Capture purity:>90%	In vivo experiments in mice	[28]
	Tumor cell membrane	Anti‐EpCAM aptamer	Cancer cell membrane substrate provides biomimetic interface with natural anti‐fouling properties and nanoscale structural compatibility for efficient CTC capture, enabling gentle release via DNA aptamer complementary strand hybridization	Capture capture: 90.2%; Capture purity: 97%	5 breast cancer patients; 5 healthy donors	[32]
CTC enrichment using a combined biomimetic membrane interface and magnetic beads	Liposome	Anti‐EGFR antibody	Enhanced cell capture efficiency through deformable antibody receptors in lipid bilayer structure that strengthen interactions with nanoscale cell surface components	Sensitivity: 5–181 CTCs/7.5 mL	7 patients with colorectal cancer	[103]
	Liposome	Anti‐EpCAM antibody and Anti‐EGFR antibody and Anti‐MUC‐1 antibody and Anti‐Her2 antibody	Lipid coating prevents magnetic core oxidation to maintain separation performance while providing high‐density antibody anchoring sites for multi‐antibody synergistic CTC capture	Capture efficiency: 82%	32 patients with multiple cancer types (14 types); 20 healthy donors	[27]
	Artificial cell membrane	Anti‐EpCAM antibody	Graphene nanosheet‐mediated simultaneous integration of biotinylated phospholipids and HSA proteins enables controllable and rapid construction of artificial cell membranes, avoiding complex detergent removal steps in traditional membrane reconstitution	Capture efficiency: 87%; Capture purity: 15–105 white blood cells per 1.5 mL	6 cancer patients; 3 healthy donors	[70]
	Neutrophil membranes	Anti‐EpCAM antibody	The approach leverages three properties of neutrophil membranes (enhanced CTC interaction, reduced WBC interaction, and soft interface) to achieve high isolation efficiency, purity, and cell viability simultaneously	Capture purity: 90.68%	20 breast cancer patients and 5 healthy donors	[30]
	Platelet‐Leukocyte Mixed Membrane	Anti‐EpCAM antibody	Simultaneous utilization of platelet‐mediated CTC recognition and leukocyte membrane‐mediated homologous repulsion for enhanced capture efficiency and purity	Capture efficiency: 91.77%; Capture purity: 96.98%	20 breast cancer patients and 5healthy donors	[36]
	Red blood cell‐platelet‐cancer cell triple‐membrane	Cell membranes derived from HepG2 cells	EpCAM‐independent	Capture efficiency: 97.6%; Capture purity: 2930 ± 18 CTCs and 44 ± 7 WBCs	Only simulated samples were used	[31]
	Genetically engineered cell membrane	EGFR scFv	Genetic engineering‐based antibody expression on cell membranes enables target synergistic recognition without chemical conjugation	Capture efficiency: 85%; Capture purity 93.5%	6 patients with multiple cancers; 3 healthy donors	[34]
	Engineering Jurkat membrane fusion hybrid vesicles	EpCAM/EGFR/Her2 scFv	Genetic engineering‐based antibody expression on cell membranes enables multi‐target synergistic recognition without chemical conjugation	Capture efficiency: 70%–85%; Capture purity: 94%	19 patients with multiple cancers; 3 healthy donors	[104]
	EV–liposome hybrid vesicles	DBCO insertion + Click chemistry	Broad‐spectrum capture of heterogeneous CTCs through bioorthogonal click chemistry	Capture efficiency >80%; WBC exclusion rate 99.99%	Mouse model of melanoma	[37]
	Tumor membrane	Anti‐EpCAM aptamer and Anti‐CDH2 aptamer	Overcoming phenotypic heterogeneity	Capture efficiency: 83.6% (MCF7 cell) and 89.4% (HeLa cell)	4 cancer patients; 5 non‐cancer patients	[106]
	MCF‐7 cell	Anti‐EpCAM Aptamer	High‐performance enrichment of rare CTCs via right‐side‐out magnetic vesicles and spatially ordered tetrahedron‐mediated aptamers	LOD of 3 cells/mL for fluorometry and 6 cells/mL for colorimetry	Only simulated samples were used	[61]
	RBC membrane	EpCAM, HER2 and EGFR	TDNs with good rigid structure, stability, and spatial localization ability on the substrate surface as a scaffold	Cell viability rate >90%, LOD of the fluorescence method <4 cells/mL; LOD of the colorimetric method <2 cells/mL	Simulated samples and mice tumor models are used	[64]
Microfluidic CTC enrichment integrating nanomaterials and biomimetic membranes	RBC membrane vesicles	FA	More than 96% of the cells remain alive	High purity: 92%; High recovery: 90%	Only simulated samples were used	[107]
	RBC membrane	Anti‐EpCAM antibody	Engineered RBC membrane interface with lateral fluidity and flexibility enables dynamic multivalent recognition for efficient, high‐viability, high‐purity CTC capture and gentle osmotic release without contamination for downstream genetic analysis	Capture efficiency: 96.5%; Detection sensitivity 90%; CTCs viability: 96.1%	20 colon cancer patients; 10 healthy donors	[89]
	White blood cell membranes	Anti‐EpCAM aptamer	Homologous exclusion reduces WBC adsorption	Capture efficiency: 91.5%; Non‐specific adsorption: 10%	Only simulated samples were used	[108]
	Hybrid membrane combining tumor membrane and white blood cell membrane	Human breast cancer cell membranes	Integration of tumor‐leukocyte hybrid membrane‐coated magnetic probes with photonic crystal microfluidics enables high‐performance CTC capture and photonic‐enhanced PTT‐PDT synergistic in situ inactivation for integrated capture‐treatment strategy	Capture efficiency: 95%	Only MCF‐7 cells in buffer	[109]
	Neutrophil membrane	Anti‐EpCAM antibody	Fast neutrophil membrane fabrication via polarization method integrated with 5‐Fu‐functionalized core‐shell fibers enables high‐capacity, high‐efficiency CTC capture with immune evasion, anti‐fouling properties, and preserved cell viability	41 cells/mm^2^ with isolation efficiency maintained at 95.7%	Only simulated samples are used	[110]

#### 4.1.1 Detection of CTCs Based Solely on Biomimetic Membranes

**FIGURE 5:**
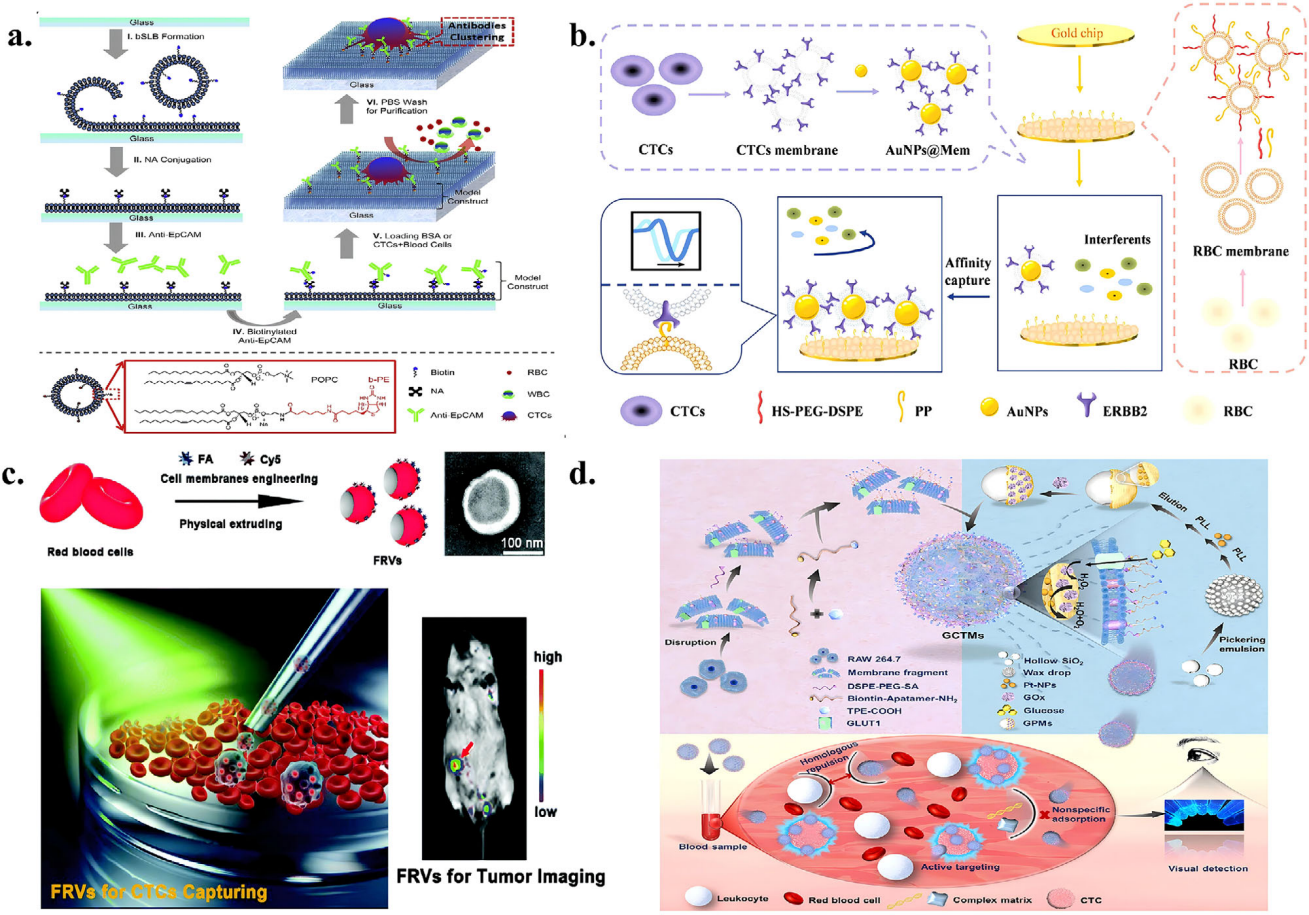
(a) Schematic illustration of the anti‐EpCAM–functionalized SLB interface for high‐purity CTC isolation. POPC/b‐PE vesicles form an antifouling supported lipid bilayer, onto which NeutrAvidin and biotinylated anti‐EpCAM are assembled for selective capture. The SLB enables ∼85% recovery and >95% purity from whole blood, while buffer rinsing removes >99.5% nonspecific leukocytes owing to its lubricating, low‐adhesion surface. Reproduced with permission [24]. Copyright 2013, Elsevier. (b) Schematic illustration of the biomimetic SPR platform using AuNPs@Mem for sensitive CTC detection. A peptide‐functionalized red blood cell membrane selectively captures ERBB2+ CTCs, while binding of AuNPs@Mem amplifies the SPR signal. The specific peptide‐protein interaction induces a ∼282 m° resonance shift, 19.7‐fold higher than the peptide‐free control, and the AuNP core provides an additional ∼4‐fold enhancement. Reproduced with permission [101]. Copyright 2025, Elsevier. (c) Schematic illustration of the folate‐functionalized erythrocyte‐vesicle platform for integrated CTC isolation and tumor imaging. FRVs enable tumor‐specific capture with minimal macrophage uptake—five orders lower than MACS. In whole blood, FRVs achieved >95% capture efficiency and >90% purity, while maintaining high viability of isolated CTCs. Reproduced with permission [28]. Copyright 2019, Royal Society of Chemistry. (d) Schematic illustration of the aptamer‐functionalized, whole‐cell biointerface for selective CTC capture and gentle release. A homologous MCF‐7 monolayer modified with BSA spacers and anti‐EpCAM aptamers enables efficient capture (∼90%) and reversible release (>90% within 30 min), with <0.25% WBC adhesion. The optimized platform achieved ∼97% purity in spiked samples and identified 0–4 CTCs/mL in patient blood. Reproduced with permission [102]. Copyright 2025, Wiley‐VCH.

Biomimetic membranes can mimic the natural cell membrane environment, preserving complex lipid and membrane protein components to achieve multivalent, multi‐point recognition of CTCs, thereby significantly enhancing capture sensitivity and specificity. Due to their high similarity to natural membranes, strong controllability, and excellent biocompatibility, lipid bilayers can incorporate various recognition molecules within a stable self‐assembly environment, enabling gentle and specific capture of CTCs. Wu et al. constructed an anti‐EpCAM‐functionalized surface based on SLB (Figure 5a) [24]. This platform effectively leveraged the biocompatibility of SLBs to reduce non‐specific adhesion of blood cells (BSA adsorption was only approximately 14 ng/cm^2^), achieving capture efficiencies exceeding 97% and 72% for HCT116 and Colo205 model CTCs, respectively, in spiked whole blood models. It also removes 99.5% of white blood cells via mild flow, significantly enhancing capture purity. More importantly, extremely low counts of CTCs captured on this surface (initially only 10 cells) maintain sustained proliferation in vitro, fully validating its unique advantage in preserving the viability of rare cells. Another strategy against non‐specific adsorption involves phosphocholine interfaces. Li et al. chemically engineered a CMMS to more precisely mimic natural cell membrane properties [22]. This surface constructs an inert interface through high‐density phosphatidylcholine groups to resist non‐specific adsorption of blood cells and plasma proteins, while combining folate (FA) and RGD peptide ligands to achieve active targeting of tumor cells. Experimental results demonstrate the CMMS surface exhibits exceptional anti‐biofouling capability, achieving over 99.999% rejection of blood cells. Simultaneously, in simulated whole blood samples spiked with HeLa cells, it captures tumor cells with 91.5% efficiency and nearly 100% purity. While these strategies provide chemical anti‐fouling properties, they still lack natural proteins and complex modifications.

RBCMs not only contain phospholipid bilayers but also retain intact components such as transmembrane proteins, glycan modifications, and cholesterol, endowing them with natural resistance to immune clearance, phagocytosis, and circulatory stability. Wu et al. combined the anti‐fouling properties of RBCMs with electrochemical analysis technology to construct a highly sensitive ratio‐type electrochemiluminescence (ECL) biosensor [100]. This sensor utilizes RBCMs as a fouling‐resistant interface, combined with graphitic phase carbon nitride (C_3_N_4_) as an internal standard for self‐calibration. RBCM modification significantly enhances the hydrophilicity of the sensor interface, resulting in signal inhibition rates below 5% in complex serum matrices. The sensor achieves linear detection of MCF‐7 cells across a broad concentration range 10 cell/mL–5×10^6^ cell/mL, with a detection limit as low as 3 cells/mL. The ratio signal exhibits excellent linear correlation with the logarithm of cell concentration. Validation using human serum samples demonstrated recovery rates ranging from 97.8% to 103.4%, confirming its potential for clinical application. Liu et al. further developed a SPR sensing platform based on AuNPs@Mem signal enhancement (Figure 5b) [101], integrating a biomimetic RBCM interface with DSPE‐PEG‐SH and palmitoylated peptide (PP) to form an RBCM‐PPP anti‐fouling recognition assembly. This assembly not only inherits the low immunogenicity and inherent anti‐fouling properties of the red blood cell membrane but is also firmly anchored to the SPR chip surface via Au‐S bonds. It relies on the palmitoylated peptide to achieve specific recognition of the ERBB2 protein on AuNPs@Mem. Leveraging the signal amplification effect of AuNPs@Mem, the SPR response of this platform is approximately 6.4‐fold higher than that of bare CTCs (detection limit: 868.11 particles/mL) and exhibits outstanding selectivity for ERBB2‐positive CTCs, which is about 13‐fold higher than that for other interferents.

Although the biomimetic properties of red blood cell membranes are enhanced, natural membrane functions may be partially restricted or lost. In contrast, autologous RBC vesicles retain intact membrane proteins and glycan modifications. Therefore, Chen et al. developed a biomimetic nanodiagnostic platform based on autologous red blood cell vesicles (FRVs) (Figure 5c) [28]. This platform enables specific recognition and fluorescent imaging of tumor cells by simultaneously modifying the erythrocyte membrane with the targeting molecule FA and the fluorescent probe Cy5. Key results demonstrate FRVs exhibit exceptional capture efficiency (>95%) and purity (>90%) for CTCs in whole blood samples in vitro, without impairing CTC viability or proliferation. In mouse models, the platform successfully achieved precise tumor tissue targeting and fluorescence imaging, validating its in vivo application potential.

Unlike the aforementioned strategy based on the anti‐fouling properties of RBCMs, another biomimetic membrane approach leverages the highly targeted cancer cell membrane interface to enhance the specificity of CTC enrichment. Ding et al. developed a mild‐release CTC capture platform utilizing the homophilic adhesion properties of cancer cell membranes [32]. This platform employs MCF‐7 cancer cell membranes as a substrate. A DNA aptamer targeting EpCAM protein is modified onto the membrane surface via a BSA linker arm to achieve specific CTC capture. Gentle release is then accomplished by adding complementary DNA strands for competitive binding. Experimental results demonstrate over 90% capture efficiency for MCF‐7 cells, release efficiency ranging from 73% to 92%, and non‐specific adhesion rates below 0.25% for non‐target cells. Crucially, this technology has undergone clinical validation, successfully capturing and identifying CTCs from blood samples of five breast cancer patients, proving its clinical applicability.

Biomimetic membranes possess excellent selective filtration properties in addition to reducing nonspecific adsorption. The phospholipid bilayer blocks small hydrophilic molecules while glucose transporter 1 (GLUT1) facilitates specific glucose transport. Recently, Yang et al. developed leukocyte membrane‐coated filtrable micromotors (GCTMs) for rapid CTC capture and detection (Figure 5d) [102]. The system features Janus‐structured hollow silica microspheres with glucose oxidase (GOx) and platinum nanoparticles selectively immobilized on one hemisphere, coated with functionalized leukocyte membranes. Glucose enters the intramembrane cavity via GLUT1 and is catalyzed by GOx to generate hydrogen peroxide, which is subsequently decomposed by platinum nanoparticles to produce oxygen. The asymmetric catalytic distribution creates a concentration gradient driving autonomous motion. By confining enzymatic reactions within the membrane interior, the design prevents flow field interference with aptamer recognition on the outer surface. Aptamers conjugated with TPE‐COOH form aggregation‐induced emission (AIE) probes that generate intense fluorescence upon tumor cell capture. GCTMs achieved efficient MCF‐7 cell capture within 1 min and maintained stable performance across complex matrices, including whole blood, with sensitivity reaching single cell detection. The platform successfully detected CTCs in tumor‐bearing mice, with captured cells retaining viability for subsequent culture.

Biomimetic membrane‐based CTC detection methods share the common advantage of significantly enhancing biocompatibility and resistance to non‐specific adsorption in complex blood environments by leveraging the structural properties of natural cell membranes. For instance, synthetic lipid bilayer systems effectively suppress non‐specific adhesion through biomimetic surface engineering, while red blood cell membrane‐coated platforms leverage their outstanding intrinsic anti‐fouling properties. Combined with sensing technologies such as ECL, these systems achieve ultrahigh‐sensitivity detection. Furthermore, cancer cell membrane‐based approaches exploit homotypic adhesion properties to achieve efficient capture and gentle release of CTCs. These methods generally enable gentle handling and preservation of captured cell viability, ensuring reliability for downstream analysis. However, these approaches also present limitations. For instance, although some methods demonstrate outstanding in vitro performance, their stability and universality in large‐scale clinical applications require further validation.

#### 4.1.2 CTC Enrichment Using a Combined Biomimetic Membrane Interface and Magnetic Beads

**FIGURE 6:**
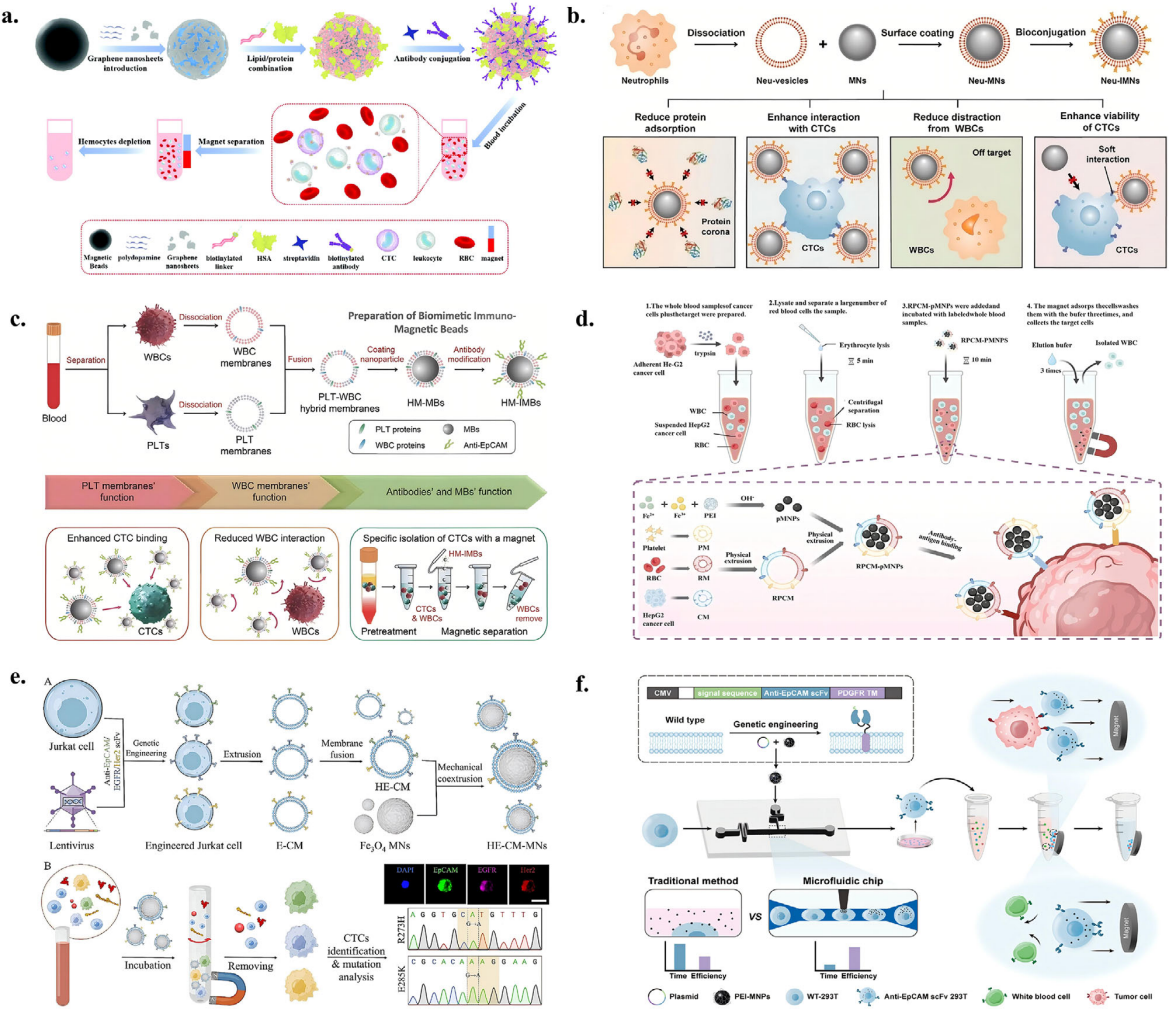
(a) Schematic illustration of the artificial cell membrane–camouflaged immunomagnetic nanoparticle (AIMNP) platform for high‐purity CTC enrichment. A synthetic lipid–HSA bilayer provides strong antifouling while maintaining antibody accessibility, enabling efficient capture (87%) and a detection limit of 7 cells/0.5 mL. The membrane camouflage removes 99.992% of leukocytes, allowing isolation of 19–685 CTCs from patient blood with minimal background. Reproduced with permission [70]. Copyright 2022, Royal Society of Chemistry. (b) Schematic illustration of the Neu‐IMN platform for efficient CTC isolation. Neutrophil‐membrane camouflage provides strong antifouling and enhances CTC recognition, maintaining >95% capture efficiency in plasma and reducing nonspecific leukocyte uptake. In spiked blood, Neu‐IMNs achieved 96.8% capture efficiency and ∼90.7% purity. Clinically, the platform isolated CTCs in 95% of breast cancer patients, enabling downstream mutation analysis. Reproduced with permission [30]. Copyright 2022, Elsevier. (c) Schematic illustration of the HM‐IMB platform for enhanced CTC isolation. Hybrid platelet–leukocyte membrane coating improves tumor binding while reducing nonspecific leukocyte uptake. With anti‐EpCAM functionalization, HM‐IMBs achieve ∼95% capture efficiency and markedly lower WBC contamination. In clinical samples, the platform isolated high‐purity CTCs in 19/20 breast cancer patients, enabling accurate PIK3CA mutation analysis. Reproduced with permission [36]. Copyright 2018, Wiley‐VCH. (d) Schematic illustration of the RPCM‐pMNP platform for synergistic CTC isolation. Magnetic nanoparticles cloaked with a tri‐hybrid membrane (RBC–platelet–cancer cell) combine long circulation, tumor homing, and homologous targeting. The hybrid coating prevents aggregation and enhances capture via integrin‐ and homotypic adhesion–mediated interactions. In spiked blood (∼5 cells/mL), RPCM‐pMNPs achieved 97.6% capture efficiency with minimal WBC contamination (∼44 cells). Reproduced with permission [31]. Copyright 2025, Royal Society of Chemistry. (e) Schematic illustration of the JE‐CM‐MN platform for specific CTC capture. Magnetic nanoparticles are cloaked with genetically engineered Jurkat cell membranes displaying anti‐EGFR scFv, enabling high‐affinity recognition (Kd=13.8 nM, ∼100‐fold stronger than native membranes). The leukocyte‐derived coating minimizes nonspecific WBC binding, achieving 93.5% capture purity. Clinically, JE‐CM‐MNs successfully isolated CTCs from all six cancer patient samples. Reproduced with permission [104]. Copyright 2024, BioMed Central. (f) Schematic illustration of the multi‐targeted hybrid membrane‐coated magnetic nanoparticle (HE‐CM‐MN) platform for capturing heterogeneous CTC subpopulations. Hybrid membranes displaying anti‐EpCAM, anti‐EGFR, and anti‐Her2 scFvs enable broad‐spectrum recognition of diverse CTC phenotypes. In mixed‐cell models, HE‐CM‐MNs efficiently captured all subtypes, outperforming single‐targeted controls. Clinically, they isolated high‐purity CTC and enabled downstream TP53 mutation analysis. Reproduced with permission [105]. Copyright 2025, Wiley‐VCH.

Traditional immune magnetic bead‐based CTC capture technology faces significant challenges due to non‐specific binding. Liposome modification creates a cell membrane‐like hydrophilic barrier and anti‐fouling interface on its surface, significantly reducing non‐specific protein adsorption while enhancing the selectivity and biocompatibility of targeted recognition. Kuai et al. constructed EGFR antibody‐conjugated magnetic liposomes (EIL) for highly efficient CTC enrichment [103]. The bilayer lipid membrane structure mimics the cell membrane, enhancing biocompatible interactions with tumor cell surfaces to significantly improve capture efficiency and reduce non‐specific protein adsorption. First, EIL exhibits excellent superparamagnetism even at room temperature. Compared to traditional EpCAM immunomagnetic beads, EIL demonstrated higher capture efficiency in clinical blood samples from seven colorectal cancer (CRC) patients, capturing 5 to 181 cells. Furthermore, the platform successfully detected KRAS gene mutations in captured CTCs, with results consistent with patient tumor tissue DNA, validating its reliability in molecular diagnostics. However, such methods remain constrained by single‐antibody loading density. Chen et al. developed a multi‐antibody‐modified magnetic immunoliposome system aimed at optimizing capture performance through high‐density anchoring of multiple antibody types [27]. This system utilizes superparamagnetic Fe_3_O_4_ as its magnetic core. By densely anchoring four key antibodies (EpCAM, EGFR, HER‐2, and MUC‐1), it significantly increases the antibody loading per nanoparticle, substantially enhancing CTC separation efficiency (80%–97.5%). Furthermore, it successfully captured and identified CTCs in clinical blood samples from 32 patients, demonstrating its potential for cancer diagnosis. Compared to liposomes, biotinylated phospholipids can directly form stable biomimetic lipid bilayers on solid surfaces. They offer advantages such as structural simplicity, controllable preparation, and ease of integration with biotin‐streptavidin systems, while avoiding issues associated with liposomes, like aggregation, rupture, and significant batch‐to‐batch variability. Zhou et al. proposed introducing biotinylated phospholipids onto magnetic nanoparticle surfaces to form biomimetic lipid layers, supplemented with anti‐fouling modifications using HSA, effectively reducing interference from non‐specific adsorption in complex blood environments (Figure 6a) [70]. Experimental results indicate that at a molar ratio of HSA to phospholipid of 13.9%, the platform achieves an average capture efficiency of 86.6%–98.5% for EpCAM‐positive tumor cell lines, with purity reaching 90.5%–96.5%. Furthermore, in 0.5 mL blood samples, the platform demonstrated a detection limit of 7 CTCs with an average capture efficiency of 87.0%. Most importantly, this technology successfully validated clinical samples, capturing 19–685 CTCs from 1.5 mL of peripheral blood in cancer patients while separating only 15–105 non‐specific white blood cells, demonstrating its potential for application in cancer liquid biopsy.

Compared to biotinylated phospholipid layers that only provide physical anti‐fouling and controllable modification sites, natural cell membranes not only retain native transmembrane proteins and glycosylation modifications—thereby conferring active recognition, immune evasion, and circulation stability at the interface—but also enable physiologically relevant capture of CTCs in complex blood environments. Leveraging the unique biological properties of neutrophil membranes, Wu et al. developed bioinspired immune magnetic nanoparticles (Neu‐IMNs) (Figure 6b) [30]. The technology employs fresh peripheral blood‐derived neutrophil membranes to encapsulate magnetic nanocores, precisely localizing anti‐EpCAM antibodies onto the membrane surface via biotin‐streptavidin chemical conjugation. The lipid bilayer structure of neutrophil membranes not only minimizes protein corona formation but, more importantly, significantly reduces non‐specific interactions with homologous leukocytes. This enhances separation efficiency (from 41.36% to 96.82%), purity (from 40.25% to 90.68%), and captured cell viability. This technology successfully isolated high‐purity, high‐viability CTCs from 19 out of 20 breast cancer patient blood samples. The isolated cells were directly usable for culture and genetic mutation analysis (PIK3CA 3140A/G), demonstrating practical application potential in non‐invasive early cancer diagnosis and precision liquid biopsy.

Compared to a single neutrophil membrane, the platelet‐leukocyte hybrid membrane combines the inherent advantages of both cell membranes. It leverages molecules such as P‐selectin on the platelet membrane to achieve efficient adhesion and chemotaxis toward CTCs, while utilizing transmembrane proteins and immune regulatory factors on the leukocyte membrane to enhance immune evasion and adaptability to the inflammatory microenvironment. This provides higher capture efficiency and circulatory stability within the complex blood environment. Rao et al. proposed a strategy of EpCAM antibody‐modified platelet‐leukocyte hybrid membrane (HM)‐based bio‐inspired immunomagnetic beads (HM‐IMBs) (Figure 6c) [36]. Experimental results demonstrate that PM‐IMBs and HM‐IMBs exhibit significantly higher capture efficiency for EpCAM‐positive MCF‐7 and HCT116 cells compared to IMBs without biofilm coating. HM‐IMBs rapidly isolate approximately 90% of tumor cells in the blood environment while substantially reducing the leukocyte background. In breast cancer patient blood samples, HM‐IMBs achieved efficient CTC capture in 19/20 cases and enabled direct downstream PIK3CA gene mutation analysis. Clinically validated, this technology demonstrates high‐purity, high‐viability CTC capture capability and strong translational potential, offering novel pathways for personalized diagnosis and therapy. Building upon this, Lin et al. further incorporated the long circulation advantage of red blood cells and the homophilic recognition properties of cancer cell membranes to construct antibody‐free red blood cell‐platelet‐cancer cell triple‐membrane camouflaged magnetic nanoparticles (RPCM‐pMNPs) for targeted CTC enrichment (Figure 6d) [31]. Experimental results demonstrate that RPCM‐pMNPs successfully inherit multiple characteristic proteins. In mouse whole blood samples, these nanoparticles exhibit outstanding performance even under low CTC concentration conditions, capturing 2930 ± 18 CTCs with high efficiency (97.6% ± 0.6%) and specificity, whereas only 44 ± 7 white blood cells were co‐captured.

In recent years, genetic engineering and biomimetic membrane technologies have increasingly been applied to CTC enrichment. Compared to antibody‐liposome/biomimetic membrane strategies relying on chemical modifications, genetic engineering enables direct expression of antibodies on natural cell membranes. This approach preserves the antibodies' native conformation and activity, supports multi‐marker synergistic recognition, and leverages the immune evasion and chemotactic properties of leukocyte membranes. Consequently, it provides a more stable, efficient, and personalized biomimetic platform for CTC enrichment. For example, Jiang et al. developed genetically engineered cell membrane‐coated magnetic nanoparticles (JE‐CM‐MNs) [34]. This technology stably overexpresses anti‐EGFR scFv on Jurkat cell membranes, effectively reducing non‐specific binding through membrane homology while enabling high‐affinity recognition of EGFR‐positive CTCs via membrane‐anchored antibodies. Results demonstrate that JE‐CM‐MNs exhibit approximately 100‐fold enhanced binding affinity for exogenous EGFR compared to controls (Kd = 13.8±2.6 nM vs. 1464.3±61.1 nM), with capture efficiency for MDA‐MB‐468 cells increasing from 25% to 85%. Moreover, the system achieved 80.4% capture efficiency in blood (R^2^ = 0.9992), and increased purity from 64.8% to 93.5%. Captured cells maintained high viability and proliferative capacity. Ultimately, this technology successfully isolated CTCs from 6/6 cancSubsequently, the team further employed genetic engineering to stably express single‐chain fragment antibodies targeting three broad‐spectrum tumor markers—EpCAM, EGFR, and HER2—on the cell surface. They then extracted membrane‐coated nanoparticles (HE‐CM‐MNs), achieving multi‐target synergistic recognition (Figure 6e) [104]. Experimental results demonstrated that HE‐CM‐MNs achieved capture efficiencies of approximately 90% for multiple tumor cell subpopulations, maintaining capture rates between 70% and 85% across 12 distinct tumor cell subpopulations. In whole blood samples, the system exhibited a capture purity of 93.9%, significantly surpassing the control group (63.5%). In testing clinical blood samples from 19 cancer patients, HE‐CM‐MNs demonstrated higher CTC capture efficiency and purity than commercial IMNs. Ultimately, the study successfully performed downstream analysis using recovered CTCs, detecting TP53 gene mutations 818G/A and 853G/A, proving its reliability for clinical applications in patient samples with 100% detection rate, while no CTCs were detected in healthy donors.

Recently, Chen et al. developed a microfluidic‐enhanced magnetic genetically engineered cell platform (Figure 6f) [105]. Plasmid‐PEI‐MNP complexes were delivered into 293T cells through 8 µm narrow channels, where synergistic mechanical squeezing and fluid focusing enhanced nanoparticle uptake, increasing transfection efficiency from 8.4% to 19.7% and magnetic separation efficiency to 87.0%. Transfected cells expressed membrane‐anchored anti‐EpCAM scFv. Using intact engineered cells as capture platforms avoided membrane extraction while ensuring correct scFv orientation, with the cellular membrane effectively shielding nonspecific binding sites. MN‐scFv 293T cells achieved 84.7% capture efficiency for rare CTCs (10 to 100 cells) and 91.9% purity in simulated blood, compared with 61.2% for commercial IMNs. Captured cells were released via 2‐min trypsin treatment with 86.9% efficiency while maintaining viability. Clinical validation demonstrated 100% detection sensitivity in 6 cancer patients with no false positives in 3 healthy donors.

**FIGURE 7:**
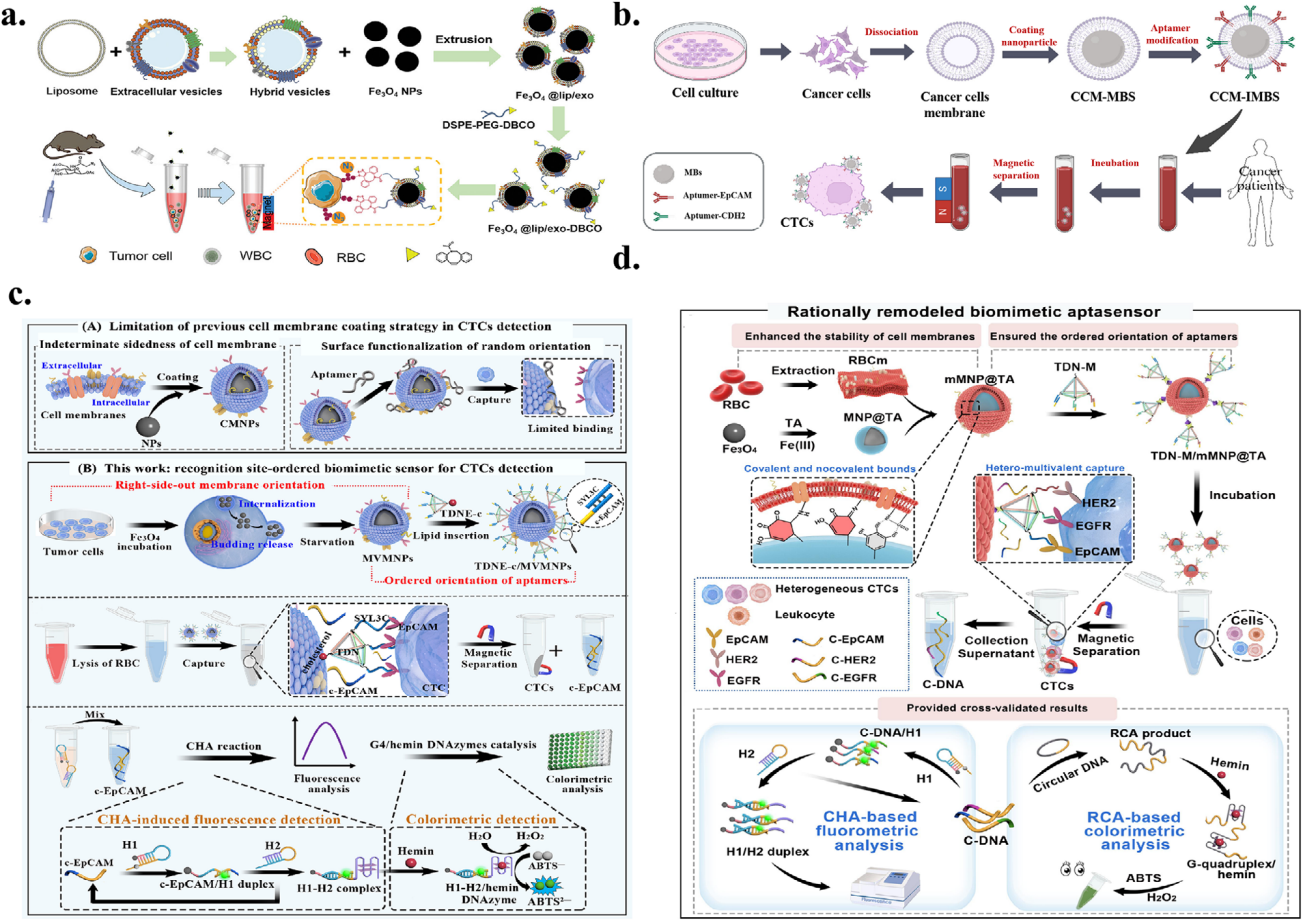
(a) Schematic illustration of the Fe_3_O_4_@lip/ev‐DBCO platform for phenotype‐independent CTC capture. Metabolic azide labeling (Ac_4_ManNAz) enables bioorthogonal click binding to DBCO‐modified, melanoma‐EV‐coated magnetic nanoparticles. The platform achieved >80% capture efficiency for both EpCAM‐positive and ‐negative cells, removed 99.99% WBCs, and detected as few as 10 cells in a lung‐metastasis mouse model. Reproduced with permission [37]. Copyright 2023, Wiley. (b) Schematic illustration of the dual‐aptamer–functionalized, cell membrane–mimetic magnetic nanoparticle (CCM‐IMB) platform for capturing heterogeneous CTC subpopulations. The biomimetic soft‐membrane interface co‐displays anti‐EpCAM and anti‐CDH2 aptamers, enabling efficient targeting of epithelial and mesenchymal CTCs (89.4% capture for mesenchymal cells). The membrane‐mimetic coating preserves metabolic integrity, and clinical assays detected CTCs in all patient samples without false positives. Reproduced with permission [106]. Copyright 2024, Elsevier. (c) Schematic illustration of the tetrahedral DNA nanostructure–engineered magnetic vesicle sensor (MVMNP) for ultrasensitive CTC detection via CHA amplification. Cell‐secreted vesicles ensure correct membrane orientation, while TDN‐assembled aptamers enhance binding and signal output. The MVMNPs showed higher capture efficiency than ultrasonication‐derived controls and, with CHA activation, achieved ultra‐low detection limits of 3–6 cells/mL in blood samples. Reproduced with permission [61]. Copyright 2024, Elsevier B.V. (d) Schematic illustration of the dual‐mode biomimetic aptasensor (TDN‐M/mMNP@TA) for ultrasensitive CTC detection. TA‐stabilized RBC‐membrane magnetic nanoparticles are functionalized with TDN‐assembled multivalent aptamers to recognize heterogeneous CTCs. The sensor showed high capture efficiencies across breast cancer subtypes and, with dual amplification (CHA/RCA), achieves ultra‐low detection limits of 1–4 cells/mL (fluorescence) and 1–2 cells/mL (colorimetric). Reproduced with permission [64]. Copyright 2025, American Chemical Society.

Compared to biomimetic magnetic beads that only provide partial membrane components and surface functions, cell vesicle‐modified magnetic beads retain the complete lipid bilayer structure and natural transmembrane protein profile. This enables them to more authentically replicate the complex biological functions of cell membranes, thereby achieving higher specificity in CTC capture, better resistance to immune evasion, and stronger physiological relevance for downstream functional studies. Kang et al. ingeniously combined EVs camouflage with glycometabolic engineering. By introducing azide groups onto CCM and leveraging their specific click reaction with DBCO (dibenzocyclooctyne) groups on nanoparticle surfaces, they achieved label‐free capture of CTCs (Figure 7a) [37]. This strategy avoids dependence on specific membrane protein expression levels, offering a novel approach for universal capture of heterogeneous CTCs. Experimental results demonstrate the platform's universal and highly efficient performance across diverse cancer cell sources (including melanoma B16/B16‐F10 and breast cancer 4T1/MCF‐7), with capture rates consistently exceeding 80%. This significantly outperforms traditional immunoaffinity strategies (some achieving only 34% capture). In a melanoma mouse model, it successfully captured phenotypically heterogeneous CTCs. Demonstrating high sensitivity in human‐like blood simulant suspensions, it detected as few as 10 CTCs while effectively removing 99.99% of white blood cells from mouse blood, exhibiting exceptional specificity.

Xu et al. proposed dual‐affinity‐body‐functionalized cell membrane vesicle‐mimetic magnetic nanoparticles (CCM‐IMB) for synergistic capture of CTCs with different phenotypes (Figure 7b) [106]. CCM‐IMB inherits the homotypic targeting recognition properties and soft interface advantages of tumor cell membrane vesicles, while integrating a dual‐aptamer system targeting the epithelial marker EpCAM and mesenchymal marker CDH2. Results demonstrated that CCM‐IMB exhibited excellent capture efficiency (>83%) and specificity for target cells, effectively reducing false negatives caused by phenotypic heterogeneity. Furthermore, the platform successfully captured CTCs from peripheral blood samples of four patients with different tumor types. To evaluate the impact of CCM‐IMB capture on subsequent single‐cell metabolomics analysis, researchers performed single‐cell mass spectrometry analysis on 20 tumor cells from routine culture and 20 tumor cells captured by CCM‐IMB from simulated peripheral blood. Results showed that 1801 metabolic features were stably detected in CCM‐IMB‐captured cells, confirming that the soft interface effectively preserved cellular metabolic integrity, making them suitable for downstream metabolomics analysis.

However, dual‐adaptor systems typically rely on flexible linkers, resulting in uncontrollable spatial orientation and limiting binding sites. DNA tetrahedra achieve multivalent, directionally controllable cooperative binding through 3D rigid scaffolds, offering enhanced structural stability and functional scalability. Jia et al. developed a recognition site‐ordered biomimetic sensor (TDNE‐c/MVMNPs‐CHA) based on tumor‐derived magnetic vesicles, enabling efficient detection of CTCs (Figure 7c) [61]. The team first utilized tumor‐derived magnetic vesicles to ensure membrane proteins maintain their natural “right‐side‐out” orientation, enabling homologous targeting recognition. Simultaneously, tetrahedral DNA nanostructures ordered EpCAM aptamers to enhance binding affinity toward target cells. Results demonstrated a capture efficiency of 90.7% for MCF‐7 cells, significantly outperforming unmodified vesicles (56.0%) and ultrasonically prepared nanoparticles (40.3%). Following CTC capture, a dual‐mode (fluorescence/colorimetric) amplification detection system based on catalytic hairpin assembly (CHA) and rolling circle amplification (RCA) enabled highly sensitive detection at 3–6 CTC/mL. Furthermore, the sensor maintained over 87% activity after 10 days of storage at 4°C and demonstrated excellent interference resistance in complex blood samples (RSD < 4.1%). Building upon prior technology, the team significantly enhanced membrane coating stability by introducing tannic acid as a chemical stabilizer. They also expanded the detection strategy from a single EpCAM aptamer to multi‐target synergistic recognition involving EpCAM, HER2, and EGFR aptamers, better addressing the challenge of marker expression changes during tumor cell epithelial‐mesenchymal transition (Figure 7d) [64]. Results demonstrate capture efficiencies of 90.6%, 84.4%, and 80% for MCF‐7, SK‐BR‐3, and MDA‐MB‐231 cells, respectively, with detection limits as low as 1–4 cells/mL. The sensor detects as few as two cells in a 200 µL sample while maintaining >90% cell viability and excellent reproducibility (RSD 2.5%–3.3%). Ultimately, this technology demonstrated effective application in spiked blood samples from healthy individuals and in tumor‐bearing mouse models, proving its potential value for highly sensitive CTC detection in complex matrices and clinical translation.

The methods covered in this section achieve highly efficient and specific enrichment of CTCs in complex blood environments through the integration of biomimetic membranes with magnetic beads. Their core advantages lie in the biomimetic membrane structure, which significantly reduces non‐specific adsorption of plasma proteins and non‐target cells, substantially enhancing capture purity and efficiency; while the magnetic beads provide rapid separation capability, enabling CTC enrichment within minutes through an external magnetic field with simple operation and no requirement for complex instrumentation. Furthermore, these methods effectively preserve the viability of captured cells, laying the foundation for downstream molecular analysis. However, such platforms commonly suffer from complex preparation procedures and poor batch‐to‐batch reproducibility. Moreover, most approaches still rely on specific surface markers, potentially failing to comprehensively capture the phenotypic heterogeneity resulting from epithelial‐mesenchymal transition.

#### 4.1.3 Microfluidic CTC Enrichment Integrating Nanomaterials and Biomimetic Membranes

**FIGURE 8:**
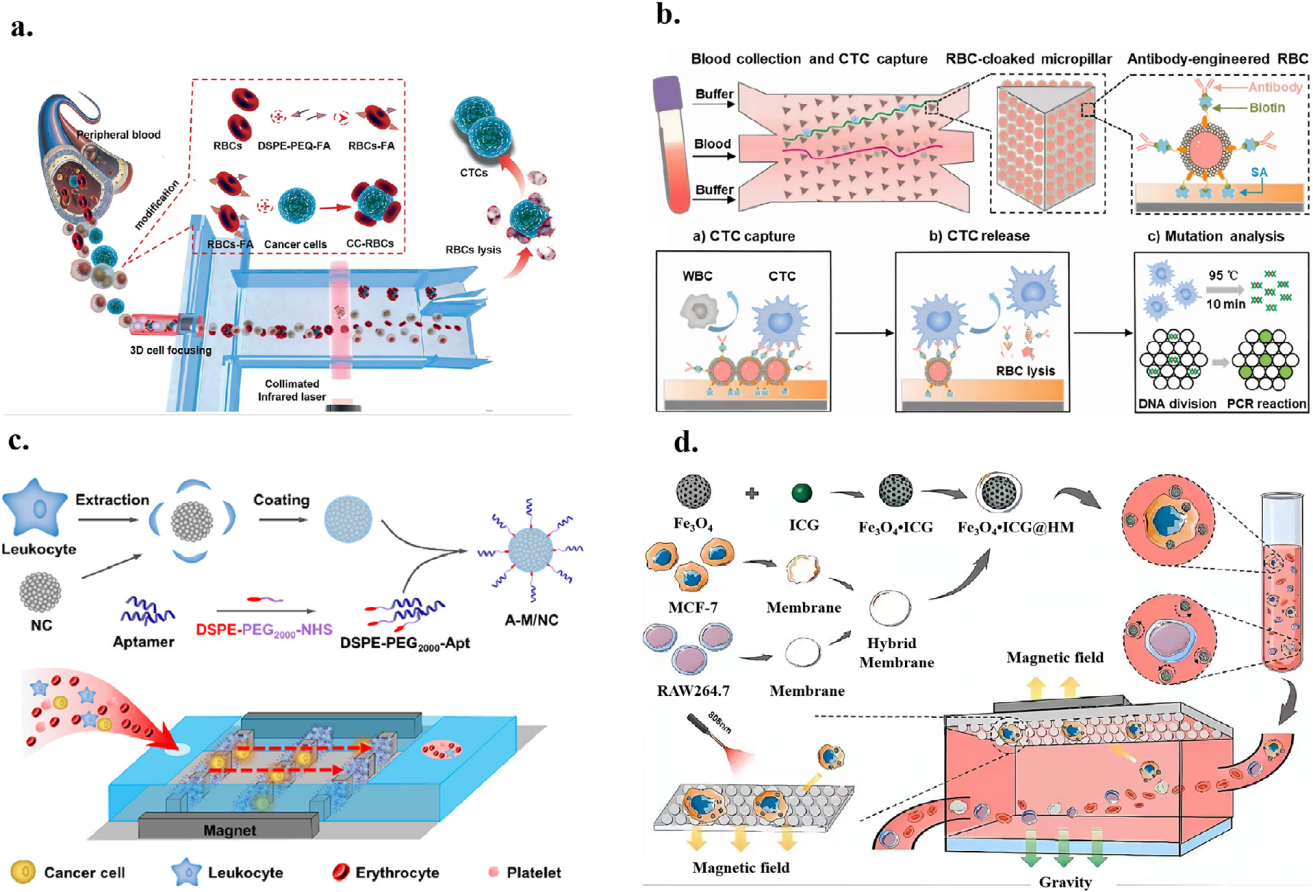
(a) Schematic illustration of the optofluidic CTC separation system using tumor‐targeted RBCs as optical amplifiers. Targeted RBCs bind CTCs to form CC–RBC complexes with enhanced optical contrast, enabling precise label‐free sorting via optical forces. Under optimized laser and flow conditions, the system achieved >90% recovery, >92% purity, a throughput of ∼2 mL/h, and preserved >96% cell viability. Reproduced with permission [107]. Copyright 2019, Royal Society of Chemistry. (b) Schematic illustration of the RBC‐Chip integrating DLD with an antibody‐functionalized soft RBC capture layer. The deformable RBC monolayer enhances CTC–surface interactions while preserving >96% cell viability. Osmotic lysis dissolves the RBC interface for gentle CTCs release (∼96.9%) without exogenous DNA contamination. In clinical testing, the chip achieved 90% sensitivity and 100% specificity. Reproduced with permission [89]. Copyright 2021, KeAi. (c) Schematic illustration of the M/NC‐integrated microfluidic system for rapid CTC detection. Leukocyte membrane–camouflaged magnetic nanoclusters (A‐M/NCs) are magnetically aligned in nickel‐patterned channels to enhance CTCs interactions while suppressing nonspecific leukocyte binding (<10%). The platform achieves >90% capture of EpCAM^+^ tumor cells and >95% detection efficiency in spiked blood, with captured cells remaining viable for downstream culture. Reproduced with permission [108]. Copyright 2019, American Chemical Society. (d) Schematic illustration of the inverted OPC‐integrated microfluidic chip combined with hybrid membrane–coated magnetic nanoprobes (Fe_3_O_4_‐ICG@HM) for CTC capture and in situ phototherapy. The inverted design reduces nonspecific sedimentation, while the tumor/WBC hybrid membrane enables dual targeting and immune evasion, achieving ∼99% specificity and >95% capture purity. Enhanced PTT/PDT effects further induce in situ photo‐inactivation of captured CTC. Reproduced with permission [109]. Copyright 2024, Elsevier.

Efficient and specific capture of CTCs from blood, along with their gentle release, remains a major scientific challenge. Integrating biomimetic membranes with microfluidic technology not only leverages the biocompatibility and anti‐fouling properties of biomimetic membranes to enhance capture specificity but also utilizes the precise fluid control of microfluidics to achieve automated, high‐throughput processing, providing a novel strategy to address this challenge. For example, Hu et al. modified homologous red blood cells with DSPE‐PEG‐FA, utilizing the specific binding of FA to tumor cells to form RBC‐CTC complexes. These complexes exhibit distinct optical constant differences (including size and average refractive index) compared to other blood cells. Through an optical microfluidic system, precise separation of modified CTCs can be achieved via laser irradiation (Figure 8a) [107]. Experimental results demonstrated that 98% of MCF‐7 cells were covered by 10–20 red blood cells, with an average of more than 5 RBCs attached per CTC. Under optimized conditions, this strategy achieved 90% recovery and 92% purity in artificial blood samples, supporting high‐throughput processing of approximately 2 mL of whole blood per hour (flow rate 100–200 µL/h), with no significant impact on cell viability as over 96% of cells remained viable.

Shen et al. developed a biomimetic microfluidic chip (RBC‐Chip) based on antibody‐engineered red blood cells (RBC‐Ab_EpCAM_), which integrates a whole‐cell red blood cell interface with a DLD patterned interaction model (Figure 8b) [89]. The chip ingeniously utilizes hydrodynamic effects to preferentially achieve collisions between large‐sized CTCs and red blood cell membranes. At the moment of collision, the lateral fluidity of the red blood cell membrane facilitates rapid antibody aggregation, enabling spatially enhanced multivalent recognition and significantly improving capture efficiency. More importantly, this technology enables non‐destructive release of CTCs through gentle lysis of red blood cells by adjusting osmotic pressure, ensuring the integrity and viability of captured cells. Experimental results demonstrated that the RBC‐Chip achieved a 96.5% capture efficiency for SW480 cells under PBS conditions, with significantly higher capture efficiency for EpCAM‐positive tumor cells compared to EpCAM‐low expressing cells, and minimal non‐specific adsorption of white blood cells. Released CTCs maintained 95.5% viability. In clinical samples, the RBC‐Chip successfully distinguished CRC patients from healthy individuals with 90% sensitivity and 100% specificity. Recovered CTCs exhibited KRAS mutations fully consistent with tissue biopsy results, demonstrating their practical value in cancer liquid biopsy applications.

Zhang et al. integrated biomimetic magnetic nanoparticles with nickel‐patterned microfluidic chips to construct a CTC enrichment platform that combines biomimetic nanomaterials with magnetically controlled microfluidics (Figure 8c) [108]. This platform utilizes leukocyte membrane fragments to camouflage magnetic nanoclusters. This camouflage not only inhibits non‐specific binding of white blood cells but also facilitates the anchoring of DSPE‐modified SYL3C aptamers, enabling specific recognition of CTCs. When the biomimetic magnetic particles are loaded into the magnetically controlled microfluidic chip, they are orderly arranged on the nickel microarray under the magnetic field, thereby enhancing topological interactions with target tumor cells. Experimental results demonstrated that within 20 min, the system achieved over 90% efficient and specific capture of multiple tumor cell types in both simulated 1:10^7^ dilution systems and untreated whole blood, while maintaining non‐specific white blood cell capture rates consistently below 10%. Regression analysis showed average detection efficiency exceeding 95% with excellent reproducibility.

Sun et al. further integrated microfluidics, magnetism, biomimetic membranes, and optical technologies to develop a unique hybrid membrane nanoprobe (Fe_3_O_4_‐ICG@HM). This probe combines the homotypic targeting properties of CCM with the anti‐fouling capability of WBCM, aiming to achieve high‐purity, efficient capture and in situ inactivation of CTCs (Figure 8d) [109]. The probe was integrated with an inverted microfluidic chip, utilizing an upward magnetic field to separate probe‐loaded CTCs from blood samples, achieving nearly 95% capture efficiency and purity. Photonic crystals integrated within the chip enhanced the photothermal and photodynamic effects of the laser. Experimental results showed that the platform achieved up to 99% positive capture signals for tumor cells while significantly reducing non‐specific adsorption of white blood cells. When captured CTCs were activated by an 808 nm laser under photonic crystal‐enhanced light fields, the synergistic photothermal therapy (PTT) and photodynamic therapy (PDT) effects mediated by the nanoprobe enabled efficient in situ inactivation of tumor cells, demonstrating the unique advantage of integrated capture‐treatment functionality of this platform.

Chen et al. developed a novel fiber pad technology based on biomimetic neutrophil membrane coating, achieving highly selective capture and functional regulation of CTCs [110]. This technology employs an alternating electric field (AEF) polarization method to rapidly prepare neutrophil membranes, followed by coaxial electrospinning to construct core‐shell structured fiber pads containing 0.6 wt% 5‐fluorouracil (5‐Fu). The outer neutrophil membrane layer achieves specific CTC capture through a streptavidin‐anti‐EpCAM antibody system while inheriting the immune evasion and anti‐fouling properties of neutrophil membranes, reducing white blood cell non‐specific adhesion rates to below 1%. The inner layer's sustained release of low‐dose 5‐Fu inhibits CTCs pseudopod extension and reduces the area occupied by individual cells, thereby significantly enhancing capture capacity. The maximum capture capacity of the fiber pad reached 41 cells/mm^2^, approximately twice that of fiber pads without 5‐Fu. In spiked blood samples, the platform demonstrated 95.7% separation efficiency in a continuous flow system at 4 mL/h with high selectivity.

The aforementioned studies demonstrate recent advances in microfluidic technologies based on biomimetic membranes and nanomaterials for CTC enrichment. Core strategies include utilizing cell membranes to camouflage magnetic nanoprobes and employing antibody‐engineered red blood cell or neutrophil membrane biomimetic fiber pads to achieve efficient, highly selective, and gentle capture of CTCs. Although these methods demonstrate outstanding performance in capture efficiency, purity, and cell viability preservation, with partial success in clinical sample analysis, limitations persist. These include complex preparation procedures, dependence on specific physical conditions, and long‐term biosafety concerns regarding nanomaterials. Consequently, the field is advancing toward multifunctional integrated platforms that combine capture, analysis, and treatment.

### 4.2 EV Detection Based on Biomimetic Membrane Interfaces

The section explores the methodologies for EV detection based on biomimetic membrane interfaces. This chapter primarily discusses how biomimetic membrane technology can be applied to various stages of EV detection and analysis. It begins with the strategies for the efficient isolation of EVs using biomimetic membrane‐based platforms and proceeds to the techniques employed for EV concentration detection. Furthermore, the section explores more advanced diagnostic applications, including the in situ detection of intravesicular RNAs and surface proteins. By covering these specific areas, this section illustrates the progression from initial sample separation to in‐depth molecular profiling enabled by biomimetic membrane‐integrated technologies (Table 3).

**TABLE 3:** Representative strategies and applications of biomimetic interface engineering in EV isolation and detection.

The purpose of the detection	Biomembrane source	Functional approach	Key advantages	Performance metrics (capture efficiency, capture purity, or detection limit)	Clinical sample validation results	References
**EV separation**	Supported lipid membranes	CD63/EpCAM antibody immobilization via biotin‐streptavidin interaction	Retention of lipid dynamics; Intrinsic non‐fouling properties	Capture sensitivity: 5×10^3^ EVs/mL (anti‐EpCAM)	Serum from 3 patients with pancreatic cancer	[23]
	Functionalized synthetic vesicles (Liposome)	Antibodies functionalized via biotin‐streptavidin interaction	Selective EV seperation under gentle centrifugation (20000 g for 30 min)	Capture capability 1.8×10^1^ ^1^ particles/mL	Human dermal interstitial fluid (ISF); FSV+EV group showed significantly larger differentially expressed proteins vs EV group	[111]
	Supported lipid bilayers decorated on magnetic beads (FluidFaceMBs)	EpCAM Aptamer, cholesterol‐terminated, anchored via hydrophobic interaction; FluidFaceMBs self‐assemble on the substrate of the HB‐Chip	Fluidity‐enhanced multivalent binding (10^5^‐fold affinity boost); Natural anti‐adsorption; Reversible magnetic release	Capture efficiency 87.8% (13.9% higher than non‐fluidic interface), with non‐specific adsorption of only 4.9%	Plasma of 20 cancer patients and 11 healthy individuals	[90]
**EV detection**	Calcein‐encapsulated liposomes	Zr^4+^ ions (acts as a bridge linker between EV and liposome phosphate groups)	Signal amplification by encapsulated reporters (calcein); Label‐free signal transduction utilizing inherent phosphate	LOD: 7.6 × 10^3^ particles/µL; Stable in 10% FBS‐containing medium.	Non‐tested on clinical samples; Feasibility shown with HeLa EVs spiked into FBS medium	[112]
	Cationic liposomes	Aptamers adsorbed on the liposome surface via electrostatic interaction	Enzyme accessibility control via electrostatic adsorption and steric hindrance; Reaction trigger by high‐affinity EV competitive aptamer dissociation	LOD: 360 particles/µL	Non‐tested on clinical samples	[69]
	PDA/Phospholipid composite Liposomes	Anti‐CD63 monoclonal antibody (conjugated via EDC/NHS)	Optical signal generation by PDA clustering/conformational change (dual colorimetric/fluorescent)	LOD (Fluorescence): 3 × 10^8^ vesicles/mL; Size change: from ∼150 nm to ∼820 nm upon EV binding	Used purified EVs from human plasma and pancreatic cancer patients (pre‐isolated via ultracentrifugation)	[113]
**EV miRNA detection**	Synthetic lipid vesicles (Liposome)	Cholesterol‐anchored Zipper DNA constructs modified on the surface and miR‐21 molecular beacons encapsulated inside	Rapid fusion kinetics via DNA hybridization; Non‐destructive and circumvents costly RNA extraction	LOD: 0.5 nM; Detection time: ∼ 30 min	Distinguished breast cancer patients from healthy individuals in plasma (4 patients vs. 4 controls)	[114]
	Liposome	Complementary dsDNAs (A'B and AB') modified on the surface via cholesterol; DNA tetrahedron‐CHA signal amplification probes encapsulated inside	Non‐destructive in situ detection and circumvents cumbersome RNA extraction	LoD: 0.24 fM	Distinguished breast cancer patients from healthy individuals and benign breast nodules woth AUC of 0.850 and 0.835, respectively (98 patients vs. 8 healthy individuals vs. 7 benign breast nodules)	[115]
	Cationic liposomes	CRISPR/Cas13a system encapsulated within liposomes	Direct detection in plasma (no pre‐extraction); Prevents RNA degradation	LOD: 1.2×10^3^ particles/mL	Distinguished breast cancer patients from healthy individuals in plasma (10 patients vs. 10 controls), AUC = 0.84	[25]
	Fusogenic nanoreactor (Liposome based)	Surface modified by hemagglutinin fusion protein; DNA‐fueled molecular machines encapsulated inside	Single‐step mix‐ and read detection; Nonenzymatic signal amplification	Detection time: ∼ 30 min	Distinguished breast cancer patients from healthy individuals in plasma (16 patients vs. 6 controls), AUC = 0.95	[116]
	Liposome	Encapsulated CRISPR/Cas13a sensing components	Amplification‐ and extraction‐free detection; Single‐EV counting for specific miRNA‐positive subpopulations	Accurate quantify miRNA‐positive EV counts using 1 × 10^8^ EVs	Distinguished ovarian cancer patients from healthy individuals (5 patients vs. 3 controls)	[180]
	Liposome	CRISPR/Cas13a sensing probes (Cas13a/crRNA/RNA reporter) encapsulated inside liposomes	Lysis‐free in situ profiling (preserves EV integrity and RNA); Signal amplification via femtoliter nanoreactors	LOD: 2.5 × 10^1^ EVs/mL	Distinguished Colorectal Cancer from non‐cancerous controls; AUC: 1.000 for miRNA‐23a and 0.8889 for miRNA‐21 (5 patients vs. 9 controls)	[117]
	Liposome	Cholesterol‐DNA tages modified on the surface and gold nanoflares (AuNFs) encapsulated inside	One‐pot assay (simultaneous tracing and profiling); Selective sorting (orthogonal barcoding); In situ profiling.	LOD: 1.2×10^5^ particles/µL (spiked plasma)	Distinguished prostate cancer (PCa) from benign prostatic hyperplasia (BPH) with 100% accuracy; Classified metastatic vs. nonmetastatic PCa with 90.6% accuracy (27 nonmetastatic PCa vs. 20 metastatic PCa vs. 2 BPH)	[118]
	Liposome	Cholesterol‐DNA tags modified on the surface and CRISPR/Cas13a components encapsulated inside	DNA‐guided orthogonal membrane fusion; Single‐vesicle miRNA profiling; Elimination of non‐target interference; In situ detection without RNA extraction	LOD: 14.7 particles/mL for miR‐153, 16.0 particles/mL for miR‐183, 23.7 particles/mL for miR‐940; Fusion efficiency: 83.3%	Distinguished PCa from healthy donors with 91.3% accuracy based on PCA‐LDA model; AUC values for three miRNAs: 0.931 (miR‐153), 0.923 (miR‐183), and 0.869 (miR‐940); Verified in clinical plasma (n = 23)	[119]
	Cancer cell membrane vesicles	Catalytic DNA machinery encapsulated inside	Homotypic recognition (phenotypic homology targeting); Subtype diagnosis; Signal amplification (RNA‐triggered)	LOD: 557 particles/mL	Specifically distinguishes estrogen receptor‐positive and triple‐negative breast cancer patients and enables disease staging	[33]
	Encoded‐targeted‐fusion beads (Lipid‐coated beads)	Recognition ligands and barcode signals modified on the surface, and molecular beacons encapsulated inside	Isolation‐free/lysis‐free direct plasma analysis; Multiplexed detection using encoded signatures; Rapid/low sample requirement	tEV capture efficiency: 77.1%; Non‐specific capture: 14.6%; tEV‐miRNA LOD: 1.4 µg/mL (equivalent to 14.6 fM); Plasma volume: 2 µL; Processing time: 2 h	Distinguished pancreatic cancer patients from healthy individuals in plasma (20 pancreatic cancer vs. 6 pancreatitis vs. 10 healthy individuals)	[120]
	Anionic liposomes	Encapsulates hairpin DNA probes targeting different biomarkers for multiplexed sensing.	Ca^2+^‐assisted "cationic bridge" mechanism for fusion; Charge repulsion to minimize non‐specific plasma interference; In situ profiling without vesicle lysis or RNA extraction	LOD: 50.3 fM for miR‐155‐5p, 0.016 fM for miR‐125b‐5p, and 72.2 fM for miR‐21‐5p.	Distinguished lymphoma patients from healthy controls; AUC:> 0.99 for triple‐miRNA combined diagnosis (48 lymphoma patients vs. 48 healthy controls)	[121]
**EV protein detection**	Supported lipid bilayers	Biotinylated SLBs conjugated with monoclonal antibodies	Label‐free high‐throughput sensing; Single‐particle sensitivity	Throughput: >10 000 events; Sample volume: <0.5 µL; Anti‐fouling: 4.5‐fold better than BSA passivation	Fingerprinted EV populations from four distinct ovarian cell lines over a panel of biomarkers	[122]
**EV multi‐dimensional biomarker detection**	Virus‐mimicking fusogenic vesicles	Co‐expressed HN and F proteins (mimicking viral envelope) on the surface and three engineered molecular beacons encapsulated inside	Extraction‐free multiple miRNA detection	EV capture efficiency 83%; LOD: 16‐28 EVs/µL for protein, 14‐22 EVs/µL for miRNA	Distinguished 30 pancreatic cancer patients from 10 benign controls; achieved 100% stage monitoring accuracy	[35]
	Cationic liposomes	Molecular beacons targeting miR‐21 encapsulated inside	Localized imaging of PD‐L1 and miR‐21 on individual EV	EV capture efficiency: ∼ 88.5%; LOD: 132 particles/µL for PD‐L1, ∼6.39 nM for miR‐21	Distinguished breast cancer patients from healthy donors and benign groups (n = 10 per group), AUC = 0.91. Predicted immunotherapy response in mouse models	[26]
	Liposome	Cas13a sensing for miRNA encapsulated inside.	Digital single‐EV analysis: concurrent protein/miRNA detection via dual CRISPR/Cas	LOD: 214 EVs/µL	Distinguished breast cancer patients from healthy donors (21 patients vs. 10 healthy individuals), AUC = 0.92	[124]
	Liposome	Molecular beacons targeting miRNA and dual‐specificity nucleases encapsulated inside	In suit miRNA detection; Super‐resolution imaging.	Single‐EV analysis; Protein signal enhanced 4.3‐fold via rolling circle amplification; miRNA signal enhanced 8.3‐fold over free molecular beacons	Distinguished cancer patients from controls and 3 categories of immunotherapy efficacy (n=86)	[125]
	Supported lipid bilayers on nanoforest	Antibody‐modified SLBs and molecular beacons targeting miRNA encapsulated inside	Efficient capture: nanovortices and large surface area; In situ co‐detection of protein and miRNA	Capture efficiency: ∼ 86.8%; LOD: 10^3^ tEVs/µL; Sample volumn: 40 µL	Distinguished pancreatic cancer, pancreatitis, and healthy individuals(38 cancer patients vs. 12 healthy individuals vs. 13 pancreatitis patients)	[126]
	Liposome	Molecular beacons targeting miRNA/mRNA encapsulated inside	Probes imported into single EV via fusion	Throughput: 384 samples/assay. Time: ∼6 h; Sample volumn: ∼90 µL	Up‐regulated expression of PD‐L1 mRNA and protein and miR‐21 in EVs derived from patients with lung adenocarcinoma (34 patients vs. 35 healthy individuals)	[127]
	Molecular beacon loaded charged‐liposome	Optimized surface charge and miRNA/mRNA probing components encapsulated inside	Digital fingerprinting: droplet‐based digital detection directly from plasma without RNA extraction	LOD: 79 EVs/µL for miR‐21, 1348 EVs/µL, and 3595 EVs/µL for EGFR L858R and T790M mutations	Detecte EGFR L858R and T790M mutations with AUC values of 1.0000 and 0.9784 in 83 lung cancer samples	[128]

#### 4.2.1 Efficient Isolation of EVs Based on Biomimetic Membrane Interfaces

**FIGURE 9:**
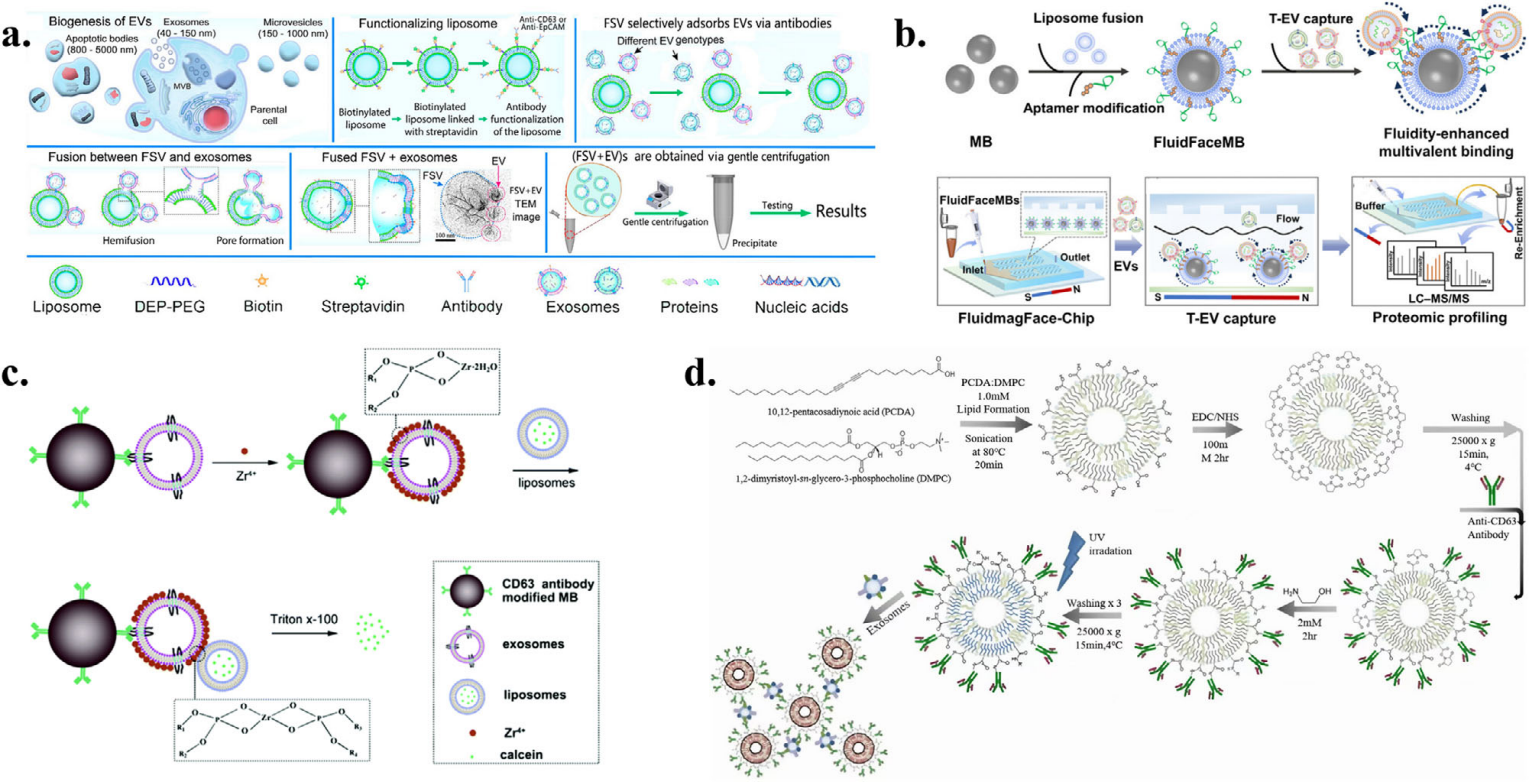
(a) Schematic illustration of the FSV platform for phenotype‐specific EV isolation from dermal interstitial fluid (ISF). A microneedle array extracts ISF, and antibody‐FSVs selectively capture EVs via membrane fusion. Optimized liposome size (100–400 nm) and low‐speed centrifugation (20 000 g) prevent structural damage seen with ultracentrifugation. The system extracts >50 µL ISF in 20 min and enables efficient EV enrichment from microliter samples for minimally invasive liquid biopsy. Reproduced with permission [111]. Copyright 2023, American Chemical Society. (b) Schematic illustration of the FluidFaceMB platform for EV capture. Magnetic beads coated with a fluid‐supported lipid bilayer enable ligand diffusion and clustering to enhance multivalent binding, boosting affinity by ∼10^5^‐fold and improving EV isolation efficiency by 13.9% with minimal nonspecific adsorption. Magnetically guided FluidFaceMBs assemble on the herringbone chip for efficient tEV capture and reversible release. Reproduced with permission [90]. Copyright 2023, Wiley‐VCH GmbH. (c) Schematic illustration of the label‐free EV detection platform based on Zr^4+^‐phosphate coordination chemistry and liposome‐mediated signal amplification. This universal capture strategy utilizes Zr^4+^ as a bridging agent to link the inherent phosphate groups on the EV surface with negatively charged liposomes, forming "EV–Zr^4+^–Liposome" complexes for magnetic separation. Stability tests confirmed the platform's resistance to complex matrix interference, even in 10% FBS. This robust, label‐free approach simplifies the workflow and achieves high sensitivity (LOD: 7.6 × 10^3^ particles/µL) for rapid EV screening. Reproduced with permission [112]. Copyright 2019, Royal Society of Chemistry. (d) Schematic illustration of the PDA vesicle–based sensor for rapid colorimetric EV detection. Antibody‐functionalized PDA vesicles bind EV surface markers (e.g., CD63), inducing vesicle clustering and a blue‐to‐red chromatic/fluorescent transition. DLS analysis confirmed aggregation, with vesicle size increasing from ∼150 to ∼820 nm upon EV binding. The assay offers dual readout with a detection limit of 3 × 10^8^ particles/mL and maintains robustness against common plasma protein interference. Reproduced with permission [113]. Copyright 2019, American Chemical Society.

To achieve specific isolation of EVs, Liu et al. proposed a capture method based on supported lipid bilayer microarrays [23]. By modifying the array surface with antibodies that recognize EVs and cancer‐specific markers, this platform enabled rapid, sensitive, and highly selective EV capture, and the nucleic acid cargo of the captured vesicles remained on the array, facilitating downstream analysis. Further experiments showed that this method achieved efficient capture in patient serum samples without additional purification steps, demonstrating its feasibility for liquid biopsy. Building on this idea, Cheng et al. proposed a functionalized synthetic vesicle (FSV) strategy based on membrane fusion mechanisms (Figure 9a) [111]. The study achieved efficient fusion‐based capture with target EVs by decorating liposomes with specific antibodies (such as EpCAM, CD63). Because the FSV+EV complexes have increased particle size, they can be separated under mild conditions (20 000 g, 30 min), avoiding the damage to EVs caused by ultracentrifugation. Experimental results showed that this method achieved nearly 100% capture efficiency before saturation, with protein concentrations significantly higher than traditional methods while maintaining vesicle integrity. Notably, this technique performed well in clinical applications: using a microneedle array, less than 50 µL of sample could be obtained from dermal interstitial fluid, and after FSV capture, the sample was ready for proteomic analysis; the resulting EVs were of higher purity and enriched in differentially expressed proteins, showing great potential for micro‐sample detection and biomarker discovery.

In addition to achieving EV separation through membrane fusion, lipid membranes can effectively reduce non‐specific adsorption and can be combined with magnetic particles to achieve specific enrichment of EVs. Niu et al. developed magnetic microspheres with a fluidic lipid bilayer (FluidFaceMB) and integrated them into a microfluidic chip (Figure 9b) [90]. This fluidic membrane interface markedly enhanced multivalent binding affinity toward target EVs (Kd improved by ∼10^5^‐fold), achieving an 87.8% capture efficiency for tEVs under microfluidic conditions—far exceeding that of ultracentrifugation (∼15%)—while maintaining minimal nonspecific adsorption (4.9%) and preserving vesicle integrity. The FluidmagFace‐Chip demonstrated strong translational potential in real clinical samples: using only 40 µL of plasma, it enabled highly selective enrichment of EVs and reliable detection of PD‐L1‐positive EVs. Its reversible fluidic interface further supported high‐purity EV release and re‐enrichment, making the platform compatible with downstream proteomics and other analytical workflows. Moreover, the system exhibits excellent scalability, providing a robust foundation for applying EV‐based liquid biopsy in cancer precision diagnosis and therapeutic monitoring.

Existing biomimetic membrane‐based EV separation techniques have demonstrated remarkable advantages, overcoming the limitations of traditional methods in terms of efficiency and vesicle integrity. The FSV strategy proposed by Cheng and Liu leverages membrane fusion mechanisms to achieve efficient and specific capture of exosomes, showing great potential in the analysis of small‐volume clinical samples. Niu and colleagues constructed magnetic microspheres with a fluid lipid bilayer, using the lateral mobility of aptamers to markedly improve capture efficiency and specificity. Furthermore, although these methods have made breakthroughs in capture efficiency, selectivity, and vesicle integrity protection, several challenges remain. These include handling complex clinical samples, achieving large‐scale standardized preparation, and ensuring membrane fusion specificity. Further optimization will be required to enable broader clinical application.

#### 4.2.2 EV Concentration Detection Based on Biomimetic Membrane Interfaces

In addition to EV isolation, biomimetic membrane‐based strategies have also been applied to EV detection. Wang et al. employed phosphate–Zr^4^^+^ coordination chemistry, using Zr^4^^+^ as a bridge to connect the native phospholipids on the EV surface with negatively charged liposomes, forming an “EV–Zr^4^^+^–liposome” complex. Combined with magnetic bead separation and liposome‐mediated signal amplification, this enabled highly sensitive, label‐free detection [112]. The method's detection limit is as low as approximately 7.6×10^3^ particles/µL, and it maintains stable performance even in culture medium containing 10% FBS, demonstrating its applicability in complex environments. However, it has not yet been validated on clinical samples, and its ability to recognize specific membrane proteins is limited, so multiplexed detection and molecular characterization remain challenging (Figure 9c).

Subsequently, Wang et al. designed an aptamer‐modified liposome system [69]. This system uses electrostatic interactions between the positive charge of the liposomes and the DNA aptamers to prevent nonspecific extension by terminal deoxynucleotidyl transferase (TdT). When target EVs are present, the aptamers dissociate from the liposomes and are extended by TdT, forming G‐quadruplexes that bind ThT to produce a fluorescent signal, thereby achieving amplified detection. Results show that this method can detect EVs in a single system without separation steps, with a detection limit as low as 360 particles/µL and sensitivity superior to ELISA. However, the performance is highly sensitive to the electrostatic adsorption efficiency and stability of the aptamer–liposome assembly, which can be disrupted by salts, serum proteins, or sequence‐dependent charge variations, thereby compromising assay reproducibility.

To enable multimodal detection and enhance readout flexibility, Kim et al. developed a label‐free exosome analysis platform based on polydiacetylene (PDA)–phospholipid composite liposomes. By conjugating anti‐CD63 antibodies onto the PDA vesicle surface, binding of exosomes induces conformational perturbations in the PDA backbone, triggering a pronounced blue‐to‐red chromatic transition accompanied by fluorescence enhancement, thereby enabling dual colorimetric and fluorescent readouts (Figure 9d) [113]. Upon interaction with EVs, the PDA vesicles expanded from ∼150 to ∼820 nm and formed clustered structures, achieving a detection sensitivity of 3 × 10^8^ particles/mL while maintaining signal stability in the presence of common interferents such as IgG, BSA, and fibrinogen. However, the method requires ultracentrifugation‐based EVs pre‐isolation from plasma and therefore cannot be directly applied to raw clinical samples.

In summary, Zr^4+^‐phosphate coordination chemistry, aptamer‐modified liposomes, and functionalized PDA vesicles represent three distinct and emerging strategies for EV detection. The Zr^4+^‐phosphate method leverages universal membrane phospholipid affinity for high‐sensitivity, label‐free concentration detection, but inherently sacrifices specificity and molecular characterization. The aptamer‐modified liposome/TdT system is technically elegant, achieving superior sensitivity and providing membrane protein molecular fingerprints. However, its core mechanism relies on DNA dissociation driven by competitive binding, making it highly susceptible to electrostatic instability and compromised reproducibility in complex biological matrices. Finally, functionalized PDA vesicles enable a simple, label‐free detection with dual chromatic/fluorescent readouts via antibody‐specific clustering. While suitable for single‐target analysis, this liquid‐phase sensor is primarily validated using pre‐isolated EVs, posing challenges for direct application in raw plasma.

#### 4.2.3 In Situ Detection of EV miRNA and Protein

**FIGURE 10:**
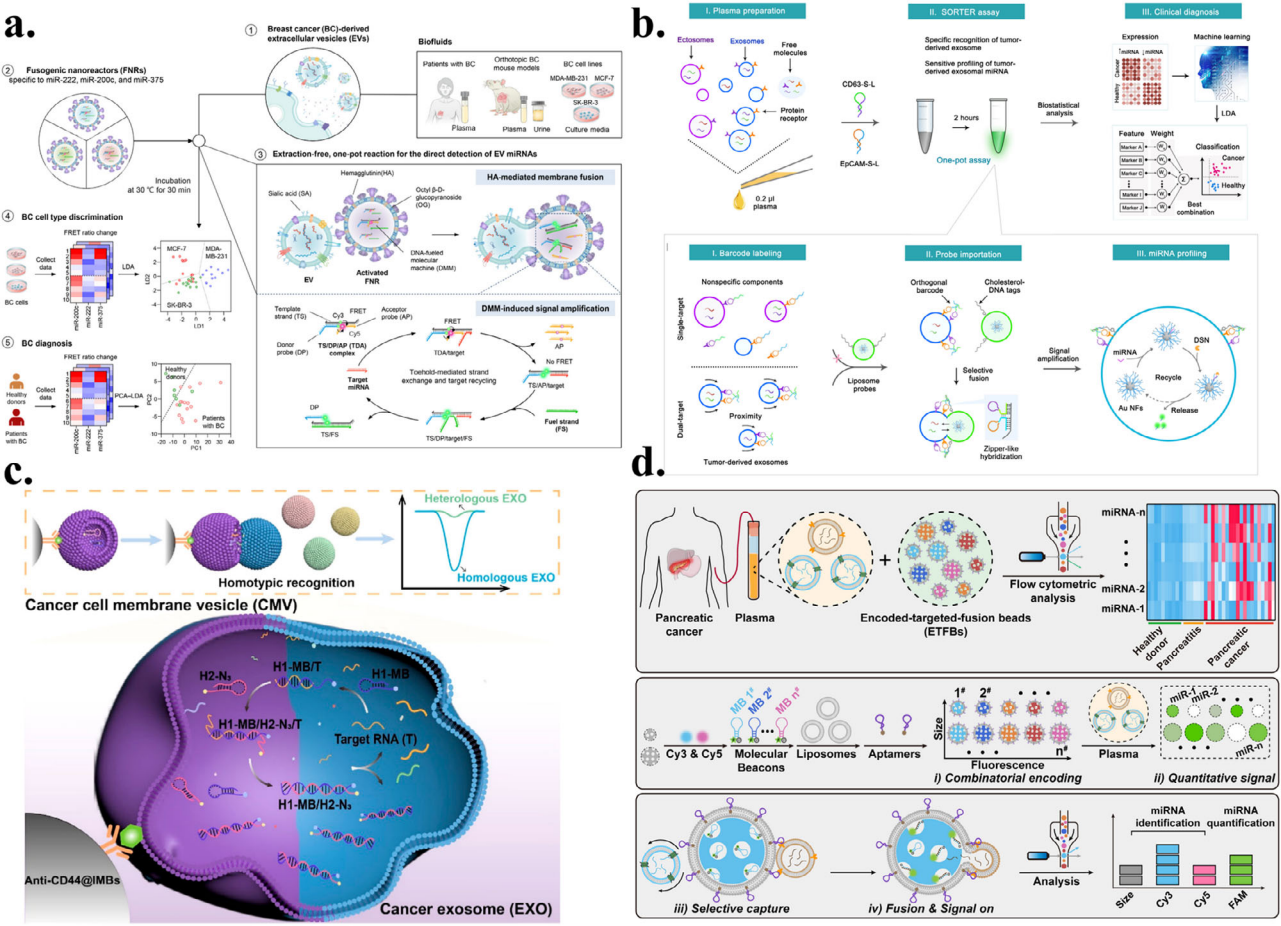
(a) Schematic illustration of the all‐in‐one FNR for in situ EV miRNA detection. HA‐mediated membrane fusion delivers DNA molecular machines into EVs, initiating a strand‐displacement cascade for signal generation. In clinical plasma, FNR achieved 86.4% diagnostic accuracy, outperforming RT‐qPCR (81.6%), and reached 95.4% accuracy with LDA analysis. Reproduced with permission [116]. Copyright 2024, American Chemical Society. (b) Schematic illustration of the SORTER platform for orthogonal EV labeling and fusion‐mediated miRNA detection. Dual surface‐protein barcodes enable selective tagging of tEVs, while DNA‐mediated membrane fusion delivers DSN amplifiers into vesicles for in situ miRNA readout. The assay requires only 0.2 µL of plasma and is completed within 2 h. Incorporating LDA to identify the optimal miRNA panel enables effective detection of prostate cancer. Reproduced with permission [118]. Copyright 2023, American Association for the Advancement of Science (AAAS). (c) Schematic illustration of the cancer cell membrane–camouflaged vesicle sensor for subtype‐specific EV detection via homotypic fusion. Leveraging the “like‐fuses‐like” membrane fusion principle, the camouflaged vesicles selectively capture tEVs and activate encapsulated DNA molecular machines to generate electrochemical signals. The sensor demonstrated high sensitivity with a LOD of ∼500 particles/mL. Reproduced with permission [33]. Copyright 2022, American Chemical Society. (d) Schematic illustration of the ETFB platform for multiplexed, lysis‐free EV miRNA detection. Encoded fusion beads with aptamer targeting, SLB‐mediated fusion, and molecular beacons enable subtype‐specific EV capture and in situ signaling. Distinct bead sizes and fluorescence barcodes allow simultaneous readout of multiple miRNAs. The assay detects miR‐21 down to 14.6 fM, and a six‐miRNA panel achieves 98% diagnostic accuracy for pancreatic cancer. Reproduced with permission [120]. Copyright 2023, American Chemical Society.

EV miRNAs have important applications in liquid biopsy, but accurately detecting EV miRNAs in complex bodily fluids and avoiding interference from other sources remain core challenges. Xie et al. proposed a zipper DNA constructs (ZDC)‐mediated membrane fusion strategy that fuses artificial liposomes containing miR‐21 molecular beacons with EVs modified with complementary ZDCs, achieving in situ miRNA recognition and signal amplification (Figure 10a) [114]. This method completes detection within 30 min, has a detection limit of 0.5 nM. By combining DNA tetrahedron and CHA to replace molecular beacons and concentrate and localize the reactants, Han et al. improved the sensitivity to 0.24 fM [115]. Building on this strategy, Zhang et al. encapsulated CRISPR/Cas13a in cationic liposomes, which fuse with negatively charged EV membranes through electrostatic attraction, enabling highly specific miRNA detection [25]. The platform reached a 1.2 × 10^3^ particles/mL detection limit, about 10^3^‐fold more sensitive than molecular beacons. Plasma validation clearly distinguished BC patients from healthy controls (AUC = 0.84), demonstrating strong clinical potential. To enhance membrane fusion specificity, Park et al. drew inspiration from the mechanism by which influenza viruses enter host cells and constructed an all‐in‐one fusogenic nanoreactor (FNR). This system employs HA to induce selective membrane fusion between the FNR and EVs, thereby delivering the encapsulated DNA‐fueled molecular machines (DMMs) into the EV lumen and triggering a nonenzymatic signal amplification. As a result, the platform enables extraction‐free, lysis‐free, and amplification‐free in situ miRNA detection within 30 min [116]. Across various biological samples—including cell culture supernatants, urine, and plasma—the FNR reliably detected three BC‐associated miRNAs and distinguished breast cancer patients from healthy individuals with a diagnostic accuracy of 86.4%, outperforming conventional RT‐qPCR. Further application of linear discriminant analysis (LDA) revealed that combining three miRNAs increased the diagnostic accuracy for BC from 78.8% to 95.4%.

Despite recent progress in EV miRNA detection, distinguishing tEVs from normal vesicles remains essential for clinical translation. Hong et al. addressed this need by capturing EVs via EpCAM‐immobilized gold micropad arrays, followed by cationic liposome–mediated delivery of Cas13a, crRNA, and fluorescent probes into intact vesicles for miRNA recognition and fluorescent imaging analysis [105]. In clinical samples, EpCAM/miR‐21‐5p double‐positive EVs were significantly elevated in patients with ovarian or Müllerian tube cancer compared with healthy individuals. Using a similar strategy, Chen et al. successfully achieved a high accuracy in diagnosing CRC, with an accuracy of 94% and an AUC of 0.954 [117]. To further enhance specificity, Lei et al. developed the SORTER platform, achieving orthogonal labeling through CD63 and EpCAM dual aptamers and using DNA tags to mediate membrane fusion to deliver probes into EVs, combined with duplex‐specific nuclease (DSN)‐catalyzed amplification to detect miRNA (Figure 10b) [118]. This method requires only 0.2 µL of plasma and 2 h to complete detection. The orthogonal labeling strategy ensures that only tEVs are interrogated, effectively minimizing background signals from normal vesicles. By incorporating LDA to integrate multiple miRNA signatures, a six‐miRNA panel achieved 100% accuracy in distinguishing prostate cancer from benign hyperplasia and 90.6% accuracy in differentiating metastatic from nonmetastatic cases. Subsequently, the team further leveraged an orthogonal barcode recognition strategy, combined with CRISPR/Cas13a signal amplification technology, to construct a tEV miRNA detection platform. Research demonstrated that the Cas13a‐mediated trans‐cleavage activity significantly enhanced the fluorescence signal by 28.6‐fold. The platform exhibited outstanding performance in the clinical diagnosis of prostate cancer, with an AUC value reaching as high as 0.985 [119]. As another approach, Cao et al. developed cancer cell membrane–mimetic vesicles that cloak catalytic DNA molecular machines in breast cancer cell membranes; by homologous fusion, they selectively capture tEVs, and endogenous miRNA triggers a cascade that is converted into an electrochemical signal (Figure 10c) [33]. Using MCF‐7 membranes, the system detects ER‐positive EVs with a miR‐375 detection limit of 557 particles/mL, and the signal correlates with clinical stage. By altering membrane sources, the platform also quantifies PD‐L1 mRNA in triple‐negative breast cancer EVs, highlighting its potential as a versatile and subtype‐discriminative EV sensing strategy.

To enable multiplexed detection of EV miRNAs, Feng et al. proposed an encoded fusion strategy, constructing targeted encoded fusion beads (ETFBs) with barcodes. Aptamer‐modified surfaces selectively capture EVs, and membrane fusion is achieved using a supporting lipid bilayer to deliver molecular beacons into the vesicle interior for detection (Figure 10d) [120]. The platform distinguishes different miRNAs by a combination of bead size and fluorescence encoding, fusion events trigger signal amplification, and analysis is performed with a conventional flow cytometer. Results show high selectivity for miR‐21 in tEVs with a limit of detection of 14.6 fM, stable in plasma; it further enabled multiplexed detection of six pancreatic cancer–associated miRNAs (miR‐21, miR‐16, miR‐10b, miR‐155, miR‐1246, miR‐196a) with high concordance to qPCR (r = 0.95). In plasma from 20 pancreatic cancer patients, 6 pancreatitis patients, and 10 healthy controls, 2 µL samples were sufficient for detection, and the six‐miRNA panel achieved a diagnostic accuracy up to 98%, significantly better than single markers. The method offers clinical potential with no separation or lysis required, high sensitivity, and rapid multiplexed detection. Ma et al. developed an anionic liposome (AL)‐based membrane fusion platform for in situ multiplexed detection of miRNAs at the single‐EV level [121]. Compared to cationic liposomes, which are prone to non‐specific electrostatic interactions, ALs significantly reduce interference from background impurities in complex plasma samples through charge repulsion. Facilitated by divalent cations (Ca^2+^), ALs achieve a membrane fusion efficiency via a “cationic bridge” mechanism. By encapsulating DNA probes targeting different biomarkers within the liposomes and utilizing nanoflow cytometry (nFCM), the platform enables ultrasensitive detection of miR‐155‐5p, miR‐125b‐5p, and miR‐21‐5p with LOD as low as 50.3, 0.016, and 72.2 fM, respectively. In a validation study of 106 clinical samples, this multiplexed system achieved a >0.99 AUC in distinguishing lymphoma patients.

Beyond EV miRNAs, EV surface proteins also reliably and accurately reflect the molecular characteristics of their parent cells, making them highly valuable biomarkers for liquid biopsy. To achieve high‐fidelity profiling of these proteins, Stollmann et al. engineered a highly uniform, ultralow‐background SLB interface within a microfluidic chip [122]. Using a modular NeutrAvidin‐biotin strategy to immobilize capture antibodies, combined with large‐field spatially incoherent digital holographic microscopy, the platform enables label‐free, single‐particle, multiprotein detection. The exceptional antifouling properties of the SLB markedly improve assay sensitivity and reproducibility. The system quantitatively measures six EV membrane proteins across seven parallel channels and employs principal component analysis (PCA) to successfully distinguish EV subpopulations derived from benign versus malignant ovarian cells. The detectable range spans from single particles to ∼200 per 100 µm^2^, and binding equilibrium is reached within approximately 5 h, highlighting its potential for EV‐subtype‐based disease diagnostics.

These membrane‐fusion–based strategies for EV miRNA or protein detection enable true in situ analysis, effectively bypassing conventional EV isolation, lysis, and RNA extraction steps. This not only shortens the overall assay time but also helps prevent miRNA degradation. Most platforms further integrate signal‐amplification mechanisms—such as CRISPR systems or DNA cascade reactions—thereby enhancing detection sensitivity and reducing sample consumption to the microliter scale. However, the controllability and reproducibility of membrane fusion efficiency remain major technical challenges, potentially introducing batch‐to‐batch variability. In addition, most studies lack large‐scale clinical validation, and their cross‐cancer applicability and analytical robustness under complex disease conditions still require systematic evaluation.

#### 4.2.4 Multi‐Marker Detection of EVs on Biomimetic Membrane Interface

Given the high heterogeneity of cancers and the complexity of their molecular networks, single‐class EV biomarkers often fail to provide sufficiently robust diagnostic information. Thus, integrating EV membrane proteins and miRNAs into multimarker panels has become essential for achieving accurate tumor identification and staging. Responding to this need, Zhou et al. developed a 3D microfluidic chip that simultaneously detects EV proteins and miRNAs within one platform [35]. Using CD63 antibodies and Y‐shaped micropillars, the chip efficiently captures EVs; CD81, EphA2, and CA19‐9 are quantified through quantum‐dot antibodies, while virus‐mimicking fusion vesicles deliver molecular beacons to probe miR‐451a, miR‐21, and miR‐10b, enabling multiplexed in situ analysis. In 40 clinical plasma samples, the combined signal of four EV markers (EphA2, miR‐451a, miR‐21, miR‐10b) clearly distinguished pancreatic cancer from noncancer cases, outperforming individual markers. With LDA, benign, early‐stage, and late‐stage groups were completely separated, achieving 100% diagnostic and staging accuracy.

However, EVs exhibit substantial heterogeneity in their cellular origin, biogenesis pathways, and molecular composition. Traditional bulk EV analyses inevitably average signals from mixed subpopulations, potentially masking diagnostically relevant molecular features. In contrast, single‐EV analysis enables profiling of variations at the level of individual vesicles, which is crucial for precise disease diagnosis. Notably, early tEVs often represent only 0.01%–0.05% of total EVs; single‐EV technologies can enhance detection sensitivity by ∼1000‐fold, effectively overcoming the limitations of bulk approaches. Moreover, single‐EV analysis provides a level of molecular resolution comparable to single‐cell omics, facilitating advances in tumor biology and the discovery of novel biomarkers [123]. Zhang et al. introduced the localized imaging of tumor‐derived individual EVs (LITIE) platform, which enables concurrent profiling of membrane PD‐L1 and intraluminal miR‐21 at the single‐EV level [26]. miR‐21 is detected through liposome‐mediated delivery of molecular beacons, whereas PD‐L1 is quantified using aptamer recognition coupled with primer exchange reaction amplification. Using only 1 µL of plasma, the system reliably discriminated breast cancer patients from healthy controls, achieving an AUC of 0.91 and outperforming single‐marker measurements.

**FIGURE 11:**
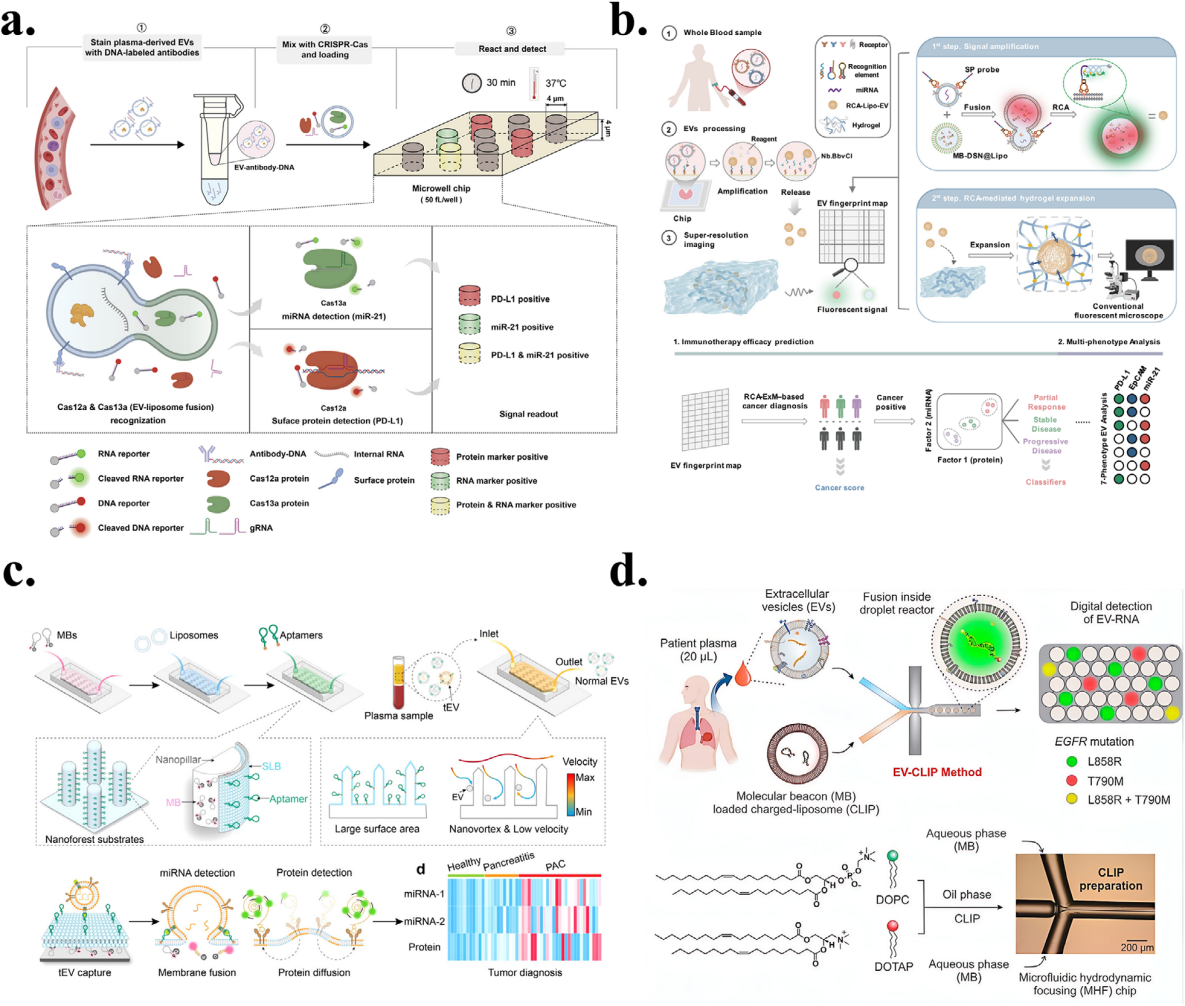
(a) Schematic illustration of the digital dual CRISPR‐Cas system (ddSEE) for concurrent single‐EV profiling. The platform utilizes a microwell array to achieve absolute quantification of surface protein (Cas12a) and inner miRNA (Cas13a via EV‐liposome fusion). This single‐molecule assay achieved high sensitivity (LOD: 214 EVs/µL), and PD‐L1+ miR‐21+ dual‐marker profiling yielded 92% diagnostic accuracy for breast cancer. Reproduced with permission [124]. Copyright 2024, American Chemical Society. (b) Schematic illustration of the RCA‐ExM platform for super‐resolution single‐EV imaging. This method combines RCA for surface protein analysis and ExM via hydrogel embedding for nanoscale resolution on standard microscopes. Internal miRNAs are simultaneously detected through molecular beacon fusion. By correlating protein and RNA co‐expression profiles (e.g., PD‐L1 and miRNAs) at the single‐vesicle level, the platform successfully stratified 86 clinical plasma samples and provided critical insights for predicting immunotherapy responses. Reproduced with permission [125]. Copyright 2025, Nature Publishing Group. (c) Schematic illustration of the FluidForest‐Chip integrating a nanoforest‐SLB for tEV capture and codetection of miRNA and protein. The 3D nanoforest enhances capture (∼86.8%) while the fluid SLB enables lateral diffusion and an “SLB dilution effect” that boosts RCA, increasing product size (∼2.6×) and signal spots (∼1.5×). The platform achieves a 3.16 fM LOD for miRNA. Reproduced with permission [126]. Copyright 2025, American Chemical Society. (d) Schematic illustration of the EV‐CLIP droplet microfluidic platform for isolation‐free, single‐EV RNA detection. Charge‐tuned liposomes fuse with individual EV inside droplets, enabling direct readout of internal RNA cargos. The optimized fusion chemistry afforded high sensitivity (LOD = 79 EVs/µL), outperforming ddPCR for rare EGFR mutations. In 83 clinical plasma samples, EV‐CLIP achieved high diagnostic accuracy (L858R: AUC 1.000; T790M: AUC 0.9784) and enabled earlier detection of drug‐resistance mutations. Reproduced with permission [128]. Copyright 2025, American Chemical Society.

Furthermore, Xu et al. developed a digital dual CRISPR‐Cas‐powered Single EV Evaluation (ddSEE) system (Figure 11a) [124]. This method utilizes antibody–DNA conjugates to convert EV surface protein signals (e.g., PD‐L1) into DNA signals, which are digitally quantified by Cas12a. Concurrently, Cas13a reagents are delivered via an EV‐liposome fusion strategy to detect intraluminal miRNAs (e.g., miR‐21), thereby minimizing interference from free‐circulating miRNAs. Operating on a microfluidic chip containing 188 000 microwells, the platform achieved single‐molecule‐level sensitivity, with a limit of detection as low as 214 EVs/µL. In clinical validation using plasma samples from 21 breast cancer patients and 10 healthy donors, the ddSEE system achieved a diagnostic accuracy of 92% based on the co‐occurrence of the PD‐L1 and miR‐21 dual markers, outperforming single‐marker measurements. To further improve resolution, Wu et al. proposed the RCA–ExM strategy, combining RCA with Expansion Microscopy (ExM) [125]. This method captures tumor EVs using an EpCAM aptamer and triggers RCA with a PD‐L1–specific hairpin probe to amplify surface protein signals, while delivering molecular beacons via liposome fusion and using a dual‐specificity nuclease to detect miRNA. Finally, hydrogel expansion enables single‐EV super‐resolution imaging. In 86 plasma samples, the technique accurately distinguished healthy individuals from cancer patients and could predict immunotherapy response, demonstrating clinical value for single‐EV multi‐component detection without expensive microscopy equipment (Figure 11b).

However, fusion‐mediated detection using vesicles like liposomes faces limitations in interfacial stability and is hampered by severe molecular crowding and steric hindrance during amplification reactions confined within nanosized tEVs, resulting in limited signal amplification efficiency. To overcome this, Gong et al. developed the FluidforestFace‐Chip, assembling a SLB onto a nanoforest substrate and integrating molecular probes to achieve efficient tEV capture and membrane‐fusion detection [126]. The unique 3D nanoforest structure enhances capture efficiency (∼86.8%) by providing a large surface area and localized low‐velocity nanovortices. Membrane fusion with the fluid SLB triggers miRNA release and hybridization detection, achieving high sensitivity (LOD = 3.16 fM). Crucially, the SLB dilution effect mitigates molecular crowding and promotes efficient RCA, enabling sensitive detection of low‐abundance proteins (e.g., PD‐L1) using a readily accessible wide‐field fluorescence microscope. This effect significantly enhanced signals, increasing protein RCA product size by ∼2.6‐fold and signal spots by ∼1.5‐fold. In clinical plasma samples (pancreatic cancer n = 38), combined detection of miR‐21, miR‐16, and PD‐L1 achieved diagnostic accuracies of 98.9% (vs. healthy individuals) and 92.9% (vs. pancreatitis patients). This technology demonstrates high‐sensitivity co‐detection under conventional microscopy conditions (Figure 11c).

In addition to EV proteins and miRNAs, EV mRNAs and genetic mutations are also important biomarkers in liquid biopsy. The Zhou team developed a high‐throughput nanobiochip system (HNCIB) [127]. This platform uses positively charged lipid nanoparticles to electrostatically capture EVs and delivers molecular beacons via membrane fusion to detect miRNA/mRNA, while combining immunofluorescent labeling to analyze proteins, and further employs TIRFM imaging and deep learning to achieve single‐EV multi‐component quantification. The system requires only about 90 µL of plasma and 6 h to complete detection, can process 384 samples in parallel, and successfully identified miR‐21, PD‐L1 mRNA, and PD‐L1 protein. In clinical validation (34 lung adenocarcinoma cases vs. 35 controls), the expression levels of miR‐21, PD‐L1 mRNA, and PD‐L1 protein in EVs derived from LUAD patients were significantly higher than those from healthy donors. In contrast, Clarissa et al. proposed the EVs‐CLIP platform [128], which achieves single‐particle RNA detection without separation or extraction by fusing charged liposomes carrying molecular beacons with EV membranes in microfluidic droplets. This method enhances fusion efficiency by tuning liposome surface charge and achieves single‐particle resolution via droplet partitioning. Experiments show its miR‐21 detection limit reaches 79 EVs/µL, and it successfully detected EGFR L858R and T790M mutations in EVs, with sensitivity superior to ddPCR. In 83 plasma samples, the platform demonstrated excellent detection of EGFR mutations (L858R: AUC = 1.000; T790M: AUC = 0.9784), and could reveal resistance mutations in advance, further proving its application value in liquid biopsy and companion diagnostics (Figure 11d).

These multi‐component simultaneous detection methods for EV analysis, by enabling synchronous detection of membrane proteins and internal nucleic acids, overcome the informational limitations of traditional single biomarkers and provide a more comprehensive molecular profile for precise tumor subtyping. Most techniques use membrane fusion strategies combined with signal amplification methods such as RCA and CRISPR systems to achieve extremely high detection sensitivity at single‐particle resolution, enabling analysis of EV heterogeneity and identification of rare subpopulations. However, the technical complexity of these methods is the main limiting factor: they require integration of multiple detection modules, raising operational barriers and potentially hindering clinical implementation. In addition, their generalizability and standardization across disease types remain to be validated, and the effective integration of multidimensional data and clinical interpretation also requires more mature bioinformatics support.

## 5 Challenges and Prospects

### 5.1 Cross‐Learning of CTC and EV Detection Technologies

As two key biomarkers in liquid biopsy, CTCs and EVs exhibit significant differences in size and abundance, yet share certain commonalities in biological structure. Both possess similar phospholipid bilayer membrane architectures with surfaces enriched in transmembrane proteins, glycoproteins, and lipid modifications, providing a common molecular foundation for membrane interface‐based recognition and capture strategies.

At the surface marker level, CTCs and tEVs partially share key molecular features. EpCAM, a classical CTC capture target, is also expressed on the surface of certain tEVs [129, 130]. Tumor‐specific receptors such as PSMA [131], MUC‐1 [132], and HER2 [133, 134] may be expressed on both CTCs and EVs in certain tumor types. Additionally, tetraspanin family members such as TSPAN1, classical EV markers, have been shown in recent studies to be similarly expressed on CTC surfaces [135]. This partial overlap in markers suggests that antibody or aptamer‐based affinity capture strategies may have cross‐platform application potential.

Orthogonal recognition strategies have shown similar developmental trends in both fields. In EV detection, Lei et al. developed the SORTER platform utilizing CD63/EpCAM dual‐aptamer orthogonal recognition to specifically sort tumor‐derived exosomes from complex plasma samples for in situ miRNA analysis [118]. In CTC detection, Li et al. further expanded this concept by constructing an EpCAM/PTK7/CD71 ternary orthogonal recognition logic gate to distinguish different tumor cell subtypes [136]. This technical convergence reflects the common need for high‐specificity multiparametric recognition in liquid biopsy.

Biomimetic membrane interface technology has demonstrated a shared trend of reducing nonspecific adsorption in both CTC and EV isolation. EV isolation requires enrichment of nanoscale vesicles from complex biological fluids while excluding interferents with overlapping sizes such as lipoproteins and chylomicrons. CTC capture faces interference from abundant background cells including leukocytes in blood, requiring high‐purity enrichment at extremely low abundance. Biomimetic membrane materials have become ideal choices for constructing high‐performance capture interfaces due to their inherent antifouling properties, biocompatibility, and membrane fluidity‐mediated dynamic ligand clustering effects. Huang et al. constructed a peptide‐engineered erythrocyte membrane‐wrapped magnetic nanoparticle biomimetic platform for EV separation and enrichment. This system improved EV capture efficiency from 46.64% to 89.22% through membrane fluidity‐mediated clustering of phosphatidylserine‐specific peptides, demonstrating excellent anti‐interference performance in PBS, serum, and complete culture medium [29]. Jia et al. developed a tetrahedral DNA nanostructure multi‐aptamer modified erythrocyte membrane‐wrapped magnetic bead biomimetic platform for CTC isolation and enrichment. This achieved 80%–90% capture efficiency for breast cancer cells of different phenotypes, with significantly reduced nonspecific adsorption of serum proteins and leukocytes after membrane coating [64]. Beyond erythrocyte membranes, biomimetic membrane strategies have expanded to include leukocyte membranes, cancer cell membranes, and hybrid membrane composite modifications, with relatively mature applications in the CTC field. These design principles of constructing low‐background interfaces based on natural cell membranes and integrating multifunctional detection may theoretically provide methodological insights for EV isolation. However, technology transfer must fully consider the fundamental differences between CTCs and EVs in scale, abundance, and biophysical properties, exploring technology migration and integrated applications through targeted engineering optimization.

### 5.2 Synchronous Analysis Technologies for CTCs and EVs

Although coordinated analysis of CTCs and EVs is crucial for precision medicine, establishing a standardized isolation workflow capable of accommodating these two vastly different biomarkers remains extremely challenging. While Leon et al. successfully achieved co‐isolation of CTCs and tEVs in metastatic CRC patients using the CellSearch system combined with ACCEPT software, with tdEV detection significantly higher than CTCs [137], this system has inherent limitations. Because CellSearch must remove plasma components before immunomagnetic EpCAM enrichment, substantial EVs are lost in the process [138]. Given this limitation, some studies have adopted stepwise processing strategies to preserve both markers, utilizing post‐centrifugation blood cell fractions for CTC isolation and plasma fractions for EV detection [139]. For example, Keup et al. combined positive immunomagnetic bead sorting with membrane affinity separation to obtain CTCs and EVs, respectively [140], while Bu et al. constructed a functionalized alginate microbead platform, achieving independent capture of both through conjugation with EpCAM and CD63, respectively [141]. Zhu et al. employed filtration combined with ultracentrifugation for CTC and EV separation [142]. However, these stepwise synchronous detection methods limit clinical translation feasibility. These approaches commonly suffer from operational bottlenecks, including multiple centrifugation steps, prolonged processing times, and dependence on frequent manual pipetting, leading to increased sample loss risk and difficulty meeting clinical testing demands. This reliance on cumbersome manual operations highlights a critical yet often overlooked challenge in the current field, namely, the absence of high‐throughput automation technologies. Without the capability to rapidly process large volumes of clinical samples within short timeframes, liquid biopsy technologies cannot achieve true clinical scalability and widespread application. Therefore, developing high‐throughput parallel processing capabilities represents an important direction for advancing combined CTC and EV detection toward clinical practice. As a proof‐of‐concept demonstration in this direction, the DUO device developed by Kang et al. successfully overcame the limitations of traditional stepwise processing [131]. As one of the few single‐platform synchronous isolation examples currently available, this study utilized radial flow design and bean‐shaped microcolumn arrays functionalized with MCAM and MCSP antibodies to successfully capture melanoma CTCs and exosomes from a single sample. The system integrated a CTCBean module for whole blood processing with an ExoBean module for plasma enrichment, thereby demonstrating excellent clinical applicability. Specifically, this platform maintained high capture efficiency for both CTCs and exosomes while sustaining processing flow rates up to 10 mL/h. This integrated strategy not only substantially reduced manual operation steps and avoided time‐consuming ultracentrifugation but also effectively resolved the contradiction between isolation purity and processing throughput, providing a powerful technical paradigm for implementing multi‐marker liquid biopsy in routine clinical settings.

### 5.3 Artificial Intelligence Applications in CTC and EV Analysis

CTCs and EVs exhibit high heterogeneity, and their detection data involve complex high‐dimensional structures, including multichannel fluorescence, super‐resolution imaging, single‐particle multiparametric features, and even multi‐omics information, making traditional empirical analysis not only inefficient and poorly reproducible but also unable to fully exploit potential biological features. AI, particularly machine learning and deep learning, with its powerful data analysis and pattern recognition capabilities, has demonstrated advantages unmatched by traditional methods in overcoming data dimensionality, deeply resolving biological heterogeneity, achieving precise classification, and enabling multimodal decision‐making. This has greatly enhanced the accuracy and efficiency of liquid biopsy analysis and has become a core driving force in translating CTCs and EVs liquid biopsy technologies from laboratory to clinic.

At the level of basic image and biological heterogeneity analysis, AI has addressed the subjectivity and inefficiency of traditional manual image analysis, significantly improving recognition accuracy at single‐cell/single‐vesicle resolution. For example, utilizing convolutional neural networks for automated CTC identification in immunofluorescence in situ hybridization images effectively reduces human bias [143]. Furthermore, for subtle differences in cell morphology, hybrid methods combining a convolutional neural network and a support vector machine can achieve high sensitivity and specificity exceeding 90% [144]. For single EV analysis, an unsupervised machine learning algorithm t‐SNE applied to high‐dimensional single EV fluorescence features acquired by multicolor single‐molecule burst analysis can identify rare EV subpopulations and resolve inflammation‐associated EVs from lipoprotein contaminants without prior labeling, achieving higher‐resolution single EV heterogeneity analysis than traditional methods [145]. Using Sobel‐operator machine learning algorithms for edge detection and colocalization analysis of single EV multichannel fluorescence images enables automated identification of tumor‐derived single EVs co‐expressing miR‐21 and PD‐L1, overcoming the limitation of traditional bulk detection in distinguishing tumor‐derived from non‐tumor‐derived EVs [26].

In clinical diagnosis and prognosis analysis, AI has been demonstrated by multiple studies to significantly improve diagnostic accuracy and prognostic prediction capability. For example, dual‐branch deep learning networks fuse CTC image features from ResNet18 and fluorescence attributes from multi‐layer perceptron for automated identification and quantitative analysis of CTC images from metastatic breast cancer patient blood. The automatically calculated CTC count with a prognostic threshold of 8 CTCs showed significant correlation with patient progression‐free survival [146]. AI models can also accurately predict breast cancer metastasis risk based on CTC molecular or phenotypic features such as epithelial‐mesenchymal transition status, achieving a high specificity of 97.56% [147]. In EV classification, using orthogonal partial least squares discriminant analysis models based on six EV marker features in a clinical training cohort containing 45 subjects, the model achieved high accuracies of 100% and 91% in distinguishing cancer patients from healthy individuals and multi‐cancer classification, respectively [148]. k‐nearest neighbor algorithm analysis of EV surface markers in a clinical cohort of 111 samples achieved accuracy exceeding 95% in distinguishing cancer patients from non‐cancer controls and approaching 90% in early lung cancer diagnosis [149]. Additionally, in multimodal analysis, AI can overcome single‐marker limitations and enable joint multi‐marker analysis. The multimodal liquid biopsy system developed by Bu et al. utilizes k‐means clustering and PCA to automatically integrate signals from CTCs, EVs, and circulating cell‐free DNA (cfDNA), generating a standardized MMLBScore with cancer discrimination AUC of 0.894, significantly superior to any single marker [141]. This model can also predict tumor T stage with AUC of 0.761 and perform risk stratification for disease‐free survival and overall survival, demonstrating the comprehensive value of AI in clinical decision‐making.

However, widespread clinical translation of AI in liquid biopsy still faces major challenges spanning technical and clinical domains. First is the issue of data standardization and model generalizability. Due to the lack of unified protocols for CTC and EV isolation and characterization, cross‐platform data heterogeneity is high, making it difficult for trained AI models to maintain robustness in new clinical environments. Second, model reliability heavily depends on the quality and scale of training data, with limited sample sizes potentially leading to overfitting. Therefore, establishing standardized databases is crucial for developing robust models. Additionally, lack of model explainability represents a core obstacle preventing AI entry into clinical decision‐making. Most current deep learning models are black boxes with opaque decision‐making processes that are difficult to gain clinician trust. Therefore, developing explainable AI algorithms and establishing verifiable connections between AI decision logic and known biological or pathophysiological mechanisms is the essential path to enhancing model biological credibility and clinical acceptance.

### 5.4 Combined Analysis of CTCs, EVs, and Other Biomarkers

Since CTCs, EVs, and other circulating biomarkers reflect different biological dimensions of information regarding tumor cell presence, molecular release characteristics, and genetic variations, a single biomarker often fails to comprehensively characterize tumor heterogeneity and dynamic evolution. Therefore, multi‐biomarker combined analysis has become an important strategy for enhancing the clinical efficacy of liquid biopsy. However, these biomarkers exhibit significant differences in measurement units, numerical ranges, and biological significance. How to effectively integrate heterogeneous data and exploit their complementary value represents a key challenge for achieving precise diagnosis and prognostic assessment. Fernandez‐Garcia et al. performed quantitative analysis of CTCs and cfDNA in blood samples from 193 metastatic breast cancer patients [150]. They employed standard deviation normalization to convert variables with different units, including CTC counts (cells/mL), cfDNA concentration (ng/mL), CA15‐3 (U/mL), and alkaline phosphatase (U/L), into dimensionless standardized continuous variables with comparable scales. The combined analysis results showed that the sensitivity for predicting disease progression reached 90%, significantly superior to using cfDNA or CTCs alone, fully demonstrating the synergistic effect of multi‐biomarker complementarity. Furthermore, the AUC of combined CTCs and cfDNA analysis for overall survival prediction was 0.81, significantly higher than traditional serum biomarkers CA15‐3 (AUC = 0.65) and alkaline phosphatase (AUC = 0.62), proving that integrating multidimensional information can overcome the performance bottleneck of single biomarkers. Similarly, Ye et al. employed a logarithmic transformation to convert CTC counts and circulating cfDNA concentration into continuous variables with approximately normal distribution, and further stratified patients into four risk groups through stratification analysis based on CTC status (positive/negative) and circulating cfDNA level (high/low). This dual‐biomarker stratification system revealed a significant risk gradient effect. Patients with both high CTCs and high ccfDNA had a 17.43‐fold higher mortality risk compared to those with low levels of both markers (HR = 17.43, 95% CI: 8.52‐35.67) [151]. This risk stratification capability far exceeded that of any single biomarker. To effectively capture nonlinear relationships and higher‐order interactions between biomarkers, Yang et al. screened the optimal combination from 14 candidate biomarkers through LASSO regression, constructing a diagnostic model for pancreatic ductal adenocarcinoma (PDAC) with 5 biomarkers (EV‐CK18 mRNA, EV‐CD63 mRNA, EV‐miR‐409, ccfDNA concentration, and CA19‐9) and a staging model with 4 biomarkers (EV‐miR‐1299, EV‐GAPDH, mutant KRAS allele fraction, and CA19‐9). This method automatically handled unit differences and weight allocation among different biomarkers through feature selection and coefficient optimization, avoiding the subjectivity of manual standardization. In an independent blinded validation set, this multi‐analyte combination model achieved 92% accuracy for PDAC diagnosis (AUC = 0.95) and 84% accuracy for detecting occult metastasis (AUC = 0.85), significantly outperforming any single biomarker or conventional imaging examination [152]. Eslami‐S et al. integrated CTC status (presence/absence as a binary variable) and PD‐L1^+^ sEV concentration (continuous variable in pg/mL) through multivariate Cox regression modeling. Using interaction analysis, they discovered that the impact of PD‐L1^+^ sEV concentration on progression‐free survival (PFS) varied with CTC status. In CTC‐negative patients, high PD‐L1^+^ sEV concentration significantly predicted worse PFS, but this association was absent in CTC‐positive patients. Further risk stratification analysis showed that patients with both CTC positivity and high PD‐L1^+^ sEV concentration had the worst overall survival (OS). This study addressed unit differences through statistical modeling and cutoff optimization rather than direct standardization, demonstrating that the complementarity of CTCs and PD‐L1^+^ sEVs was superior to any single biomarker, while ctDNA mutation detection showed no independent prognostic value [153]. This suggests that multi‐biomarker integration requires full consideration of interactive effects between biomarkers rather than simple linear superposition. Bu et al. integrated three biomarkers with different units, including CTC counts, exosomal nucleic acid quantity, and cfDNA concentration, through k‐means clustering algorithm and PCA, reducing the 3D heterogeneous data to a 2D map. The first principal component of PCA (PCA‐X) was defined as the multi‐marker liquid biopsy score (MMLBScore), equivalent to a standardized linear combination of the three biomarkers, thereby automatically resolving the unit inconsistency issue. The diagnostic accuracy of combining the three biomarkers (AUC = 0.894) was significantly superior to any single biomarker (CTC: AUC = 0.826, exosomes: AUC = 0.763, cfDNA: AUC = 0.820) and successfully predicted tumor T stage, disease‐free survival, and overall survival, while also enabling KRAS mutation detection [141]. In summary, multi‐biomarker combined analysis significantly enhances the diagnostic sensitivity, specificity, and prognostic predictive capacity of liquid biopsy by integrating complementary information from different biological dimensions. The key to data integration lies in adopting appropriate standardization methods, feature selection algorithms, statistical modeling, or machine learning approaches to address the challenges of unit inconsistency and weight allocation among different biomarkers.

### 5.5 Challenges and Prospects for Clinical Translation

Although biomimetic membrane interface‐based detection methods have demonstrated promising performance in proof‐of‐concept studies, current research remains largely limited to small sample sizes and must address several critical obstacles regarding standardization, inter‐laboratory reproducibility, and long‐term biosafety.

Current biomimetic membrane interface‐based CTC and EV detection research faces technical validation challenges including small sample sizes, insufficient standardization, and lack of inter‐laboratory reproducibility. First, existing studies have limited clinical validation sample sizes and have not yet established reliable clinical thresholds. For example, the prognostic threshold for CTC detection (CTC ≥5 per 7.5 mL blood) was established through a large‐scale multicenter study by Cristofanilli et al. involving 177 metastatic breast cancer patients [154], demonstrating that adequate sample size is crucial for establishing the clinical validity of detection methods. Second, sample processing standardization and inter‐laboratory reproducibility represent key technical validation challenges. Pre‐analytical variables can account for 75% of clinical laboratory testing errors [155]. Benchmark studies by the International Society for Extracellular Vesicles showed that blood collection tube type and processing time significantly affect sample quality and EV recovery rates [156]. This lack of standardization directly results in insufficient inter‐laboratory reproducibility. Validation testing of five CTC enrichment technologies across nine laboratories by the European CANCER‐ID consortium revealed that technologies employing automated workflows and standardized operating procedures exhibited better reproducibility [157]. Therefore, to establish the clinical utility of biomimetic membrane‐based detection methods, multicenter prospective studies enrolling hundreds to thousands of patients are needed to develop comprehensive standard operating procedures from membrane material preparation to sample detection, with robustness evaluated across different laboratories and operators through multicenter validation platforms.

Regulatory compliance represents another critical barrier to clinical translation. These diagnostic platforms will likely be classified as in vitro diagnostic medical devices (IVD), with tests used for cancer diagnosis or treatment decisions typically falling into higher risk categories requiring 510(k) clearance or premarket approval. The European In Vitro Diagnostic Regulation (IVDR) mandates more stringent clinical evidence and performance validation [158]. If these platforms serve as companion diagnostics to guide specific treatments, they require co‐development and approval with corresponding drugs, necessitating early communication with regulatory agencies to establish clear validation pathways and clinical application scenarios. Regulatory agencies such as the Food and Drug Administration (FDA) and European Medicines Agency (EMA) have established stringent standards for diagnostic products based on cell‐derived materials [159]. These regulatory requirements span multiple dimensions, including ensuring authenticity and quality control of membrane materials and EV sources, establishing standardized and reproducible manufacturing processes [160], and implementing standardized testing protocols encompassing biophysical characteristics, membrane stability, and biochemical composition assessment [161, 162].

For potential in vivo application scenarios, long‐term biosafety assessment is particularly critical yet data remain insufficient. Although no in vivo CTC or EV capture studies using biomimetic membrane interfaces currently exist, if future applications extend to real‐time in vivo monitoring, such as implantable devices, long‐term biosafety assessment will become a key consideration. Regulatory agencies, including FDA and EMA require comprehensive pharmacokinetic and biosafety assessments for such in vivo applications of cell‐derived material‐based products [163, 164]. Existing in vivo safety data for biomimetic membrane materials primarily derive from drug delivery studies. Although red blood cell membrane, platelet membrane, and tumor cell membrane‐coated nanoparticles have demonstrated good biocompatibility [165, 166, 167], biosafety assessment durations in these studies typically span only approximately one week. Clinical translation experience with plant‐derived exosomes provides valuable insights. The ginger exosome study by Zhang et al. demonstrated a design approach transitioning from short‐term animal assessment (approximately 7 days) to long‐term clinical trials (28 days), establishing a systematic framework encompassing subject screening, control design, safety monitoring, and statistical analysis [168]. This framework can serve as a reference for biomimetic membrane materials intended for long‐term or repeated in vivo CTC/EV detection.

For real‐time in vivo monitoring applications, CellCollector, as the first approved in vivo CTC capture device, provides an important regulatory standardization assessment pathway reference [169]. This device strictly adheres to ISO medical device standards, establishing a complete evaluation system from in vitro cytotoxicity assessment and animal acute systemic toxicity testing to hemocompatibility verification, with clinical safety confirmation completed in healthy volunteers and cancer patients. This stepwise safety assessment strategy, progressing from in vitro evaluation through animal toxicity testing to small‐scale clinical validation, establishes a feasible pathway from laboratory research to clinical translation for biomimetic membrane interface‐based in vivo application platforms.

## 6 Conclusion

As liquid biopsy advances, the accurate, efficient, and nondestructive detection of CTCs and EVs has become a critical imperative. Biomimetic membrane interfaces offer a promising strategy to address these needs by leveraging the natural properties of cell membranes to enhance target interactions while potentially reducing background interference. Beyond serving as a technical platform for high‐performance capture, this approach provides a shared biological and molecular foundation for the synergistic analysis of CTCs and EVs. The development of this field is gradually evolving from the isolation of single targets toward the exploration of coordinated detection strategies. As discussed in this review, the shared biological signatures and structural similarities between CTCs and EVs allow for the potential cross‐application of recognition strategies and the development of integrated isolation workflows. Furthermore, the incorporation of artificial intelligence and machine learning is emerging as a supportive tool to help manage the complexity of biological data, offering new possibilities for resolving biomarker heterogeneity and assisting clinical observation. Despite this progress, significant hurdles remain before these technologies can be effectively translated into clinical settings. Future efforts should prioritize the standardization of interface fabrication and the evaluation of these platforms in broader, multicenter studies. Bridging the gap between laboratory research and clinical implementation will require continued focus on automation and the development of more interpretable computational models. Ultimately, by fostering the synergy between biomimetic materials science and targeted data analysis, biomimetic membrane interfaces are expected to contribute to the ongoing development of precision medicine, offering valuable insights for early tumor screening and treatment monitoring.

## Author Contributions

Duo Liu and Jie Yu contributed equally as co–first authors. They performed the majority of the literature search, figure design and preparation, and drafted the main parts of the manuscript. Jingxue Li assisted with literature retrieval and contributed to partial writing. Yixue Chen provided technical support and contributed to the discussion of epidemiological and methodological aspects. Mengle Peng and Yifan Xu assisted in reference management and critical analysis of related analytical chemistry content. Yongjun Wu provided constructive suggestions on the manuscript framework. Lihua Ding and Sitian He conceived the review, critically revised the manuscript, and supervised the overall work. All authors read and approved the final manuscript.

## Funding

This work was supported by the National Natural Science Foundation of China (Grant No. 82304194), the Scientific Research Project of Colleges and Universities in Hainan Province (No. Hnky2025ZC‐7), the Academic Promotion Program of Hainan Medical University (No. XSTS2025031), the China Postdoctoral Science Foundation (Grant Nos. 2023TQ0304 and 2023M743196), the Science and Technology Project of Henan Province (Grant No. 242102311174), and the Hainan Province Clinical Medical Center.

## Conflicts of Interest

The authors declare no conflicts of interest.

## Declaration of Generative AI and AI‐assisted Technologies in the Writing Process

During the preparation of this work, we used ChatGPT 5.0 in order to improve the readability and language of the manuscript. After using this tool, we reviewed and edited the content as needed and take full responsibility for the content of the published article.

## Data Availability

The authors have nothing to report.
